# Dynamic and Stable Core Microbiota Assist Plants in Enriching Selenium and Reducing Cadmium Absorption

**DOI:** 10.1002/advs.202500862

**Published:** 2025-05-19

**Authors:** Zheng Lei, Hua Zhang, Wenju Liu, Jiandong Sheng, Huan Zhang, Yin Wang, Yanni Tang, Huaxing Wang, Cuicui Ding, Wanqi Qiao, Yonghui Zhu, Guoyin Yang, Yihan Zhang, Zhuoyi Liu, Nanyu Zhou, Chengxiao Hu, Xiaohu Zhao

**Affiliations:** ^1^ College of Resources and Environment Huazhong Agricultural University Research Center of Trace Elements Wuhan Hubei 430070 China; ^2^ State Key Laboratory of Environmental Geochemistry Guiyang Guizhou 550081 China; ^3^ State Key Laboratory of North China Crop Improvement and Regulation Baoding Hebei 071001 China; ^4^ Xinjiang Key Laboratory of Soil and Plant Ecological Processes College of Resource and Environment Xinjiang Agricultural University Urumqi Xinjiang 830052 China

**Keywords:** cadmium, dynamic microbiome, glutathione metabolism, plant development, selenium, synthetic microbial communities

## Abstract

Rhizosphere microbiome is crucial for regulating rhizosphere complex nutrient dynamics. However, mechanisms by which plants regulate rhizosphere microbes to manage nutrient availability under coexisting beneficial and harmful elements remain unclear. This study focuses on the rhizosphere microbiome of *Brassica napus* in different naturally selenium (Se)–cadmium (Cd)‐rich soils, the functionality of this rhizosphere, and the changes in the availability of rhizosphere nutrients. Microbiome analysis, metagenomics, genomic analysis, strain isolation, and functional validation are performed to investigate these relationships. Results show that a significant negative correlation is observed between the rhizosphere available Se and Cd content across the plant whole growth cycle and identified a group of core microbiota that are highly positively correlated with available Se and negatively correlated with available Cd. Genomics and metagenomics analyses reveal that the core microbiota has a higher substrate preference for amino acids related to the glutathione metabolic pathway. Key glutathione‐related‐amino acids and synthetic microbial community significantly improve the expression of glutathione anabolism and related amino acid transport genes and enhance Se uptake and reduce Cd absorption in plants grown in various Se‐Cd‐rich soils. This study provides insights into the mechanisms of root‐associated microbes responding to complex soil nutrients during plant growth.

## Graphical abstract

During different developmental stages of plants in natural Se–Cd coexisting soils, there is a group of rhizosphere microorganisms with highly dynamic abundance. The abundance variation of these microorganisms is strongly positively correlated with available Se and strongly negatively correlated with available Cd. The glutathione metabolic pathway is involved in the Se‐enrichment and Cd‐reduction processes of these microorganisms in the rhizosphere.

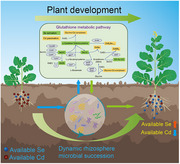

## 1 Introduction

As an extension of plant roots, rhizosphere microbes are regarded as the “second genome” of plants, promoting their growth, development, and health throughout the plant life cycle.^[^ ^1^ ^]^ Based on their unique ecological niche, rhizosphere microbes harbors more diverse and active microbial communities than bulk soil.^[^ ^1^, ^2^ ^]^ As the interface that directly interacts with plant roots, rhizosphere microbes' activation of soil nutrients directly impacts the availability of root nutrient uptake.^[^ ^1^, ^3^ ^]^ Recent studies have confirmed the role of rhizosphere microbiota in regulating elemental availability in the rhizosphere, including the activation of nutrient elements like phosphorus (P), iron, and Se, and the immobilization of heavy metal elements such as arsenic (As) and cadmium (Cd).^[^ ^3^, ^4^ ^]^ For example, under nitrogen‐deficient conditions, soybeans can recruit a group of nitrogen‐fixing microorganisms in the rhizosphere to supplement soil nitrogen.^[^ ^5^ ^]^ Under As stress, the *OsSAUR2* gene in rice regulates root secretions and the assembly of rhizosphere microbes to immobilize soil As and lower its bioavailability.^[^ ^6^ ^]^ Previous studies usually focused on single elements, yet in actual agricultural production, plants usually face soil environments where beneficial elements and heavy metals coexist. How plants achieve increased availability of beneficial elements and decreased availability of heavy metals by recruiting rhizosphere microbiota is still unclear.

Plants can recruit diverse functional microbes in the rhizosphere across developmental stages, tailored to their growth strategies, which also drives the dynamic change of rhizosphere element availability.^[^ ^5^, ^7^ ^]^ Members of the microbiota that stably exist in the rhizosphere are usually considered as the plant's core microbiome, and they provide important functions in the rhizosphere for plants throughout their development.^[^ ^8^ ^]^ However, the relative abundance of members of this stable microbiome is also highly dynamic, and specific root exudates typically drive their colonization patterns.^[^ ^3^, ^5^, ^9^ ^]^ This stable but dynamic microbial community characteristic reflects the nutrient requirements of plants at different developmental stages in the rhizosphere. For example, during the rapid growth phase of plants, there is a higher abundance of nitrogen‐fixing bacteria, which reflects the higher nitrogen demand of plants at this stage.^[^ ^5^, ^9^, ^10^ ^]^ Dynamic rhizosphere microbiota can drive dynamic changes in the availability of rhizosphere elements, not only for beneficial elements, but also for heavy metals.^[^ ^3^, ^4^ ^]^ How plants modulate rhizosphere microbes at different development stages to steer the availability of beneficial and heavy elements, and whether there's a potential link between these processes, remains largely unknown.

Recent studies have also found that many plant growth promoting bacteria (PGPB) isolated from the plant rhizosphere have difficulty in recolonizing the rhizosphere in a sustainable manner, which greatly hinders the application of synthetic microbial communities in the agricultural field.^[^ ^11^ ^]^ Exploring core microbial communities with specific functions that can sustainably colonize the rhizosphere of plants at different developmental stages is an important challenge to overcome.

Se is one of the essential elements in human beings.^[^ ^12^ ^]^ Se deficiency in the human body can increase the risk of thyroid issues, cardiovascular illnesses, male infertility, impaired immunity, and a variety of inflammatory disorders.^[^ ^13^ ^]^ However, it is estimated that ≈0.5–1.1 billion people worldwide suffer from “hidden hunger” from Se deficiency, which is due to the extremely uneven distribution of Se in the soil.^[^ ^14^ ^]^ Plants that absorb Se from the soil and enter the food chain are one of the main sources of Se intake for humans.^[^ ^15^ ^]^ However, the bioavailable fraction of Se in naturally Se‐rich regions is generally low, and only a small portion of Se in soil exists in a bioavailable fraction. Rhizosphere microbes can promote Se transformation and affect its bioavailability through oxidation, reduction, methylation, and siderophore secretion.^[^ ^16^ ^]^ Recent studies have shown that PGPB such as Bacillus, can reduce selenite or selenate to elemental Se nanoparticles and seleno–amino acids, enhancing plant Se uptake.^[^ ^4^, ^16^, ^17^ ^]^

Efficient development and utilization of the limited Se‐rich farmland soil are crucial. However, as Se‐rich soil is exploited, many surveys have also found that it not only has abundant Se but also an abundance of various heavy metal elements, notably cadmium (Cd).^[^ ^18^ ^]^ Compared with other heavy metal elements, Cd in soil is easily absorbed by plants, and it accumulates in edible plant parts, posing a potential threat to human health.^[^ ^19^ ^]^ This has led to Cd contamination in agricultural products cultivated in Se‐rich soils, thereby greatly limiting the development and exploitation of natural Se resources for agricultural production.

The transformation of soil Se into different fractions is strongly influenced by the soil microbiome, which participates in a series of processes, such as oxidation, reduction, methylation, and demethylation.^[^ ^17^, ^20^ ^]^ Inoculating with strains isolated from Se‐rich soil and Se‐hyperaccumulating plants can increase the bioavailability of soil Se and plant Se accumulation.^[^ ^4c^ ^]^ Soil Cd cannot be damaged or degraded by the soil microbiota, but its physical and chemical properties may be altered to be less toxic. Hence, the microbiota can limit plant absorption of Cd through mechanisms like extracellular complexation, intracellular accumulation, and redox reactions.^[^ ^21^ ^]^

**Figure 1:**
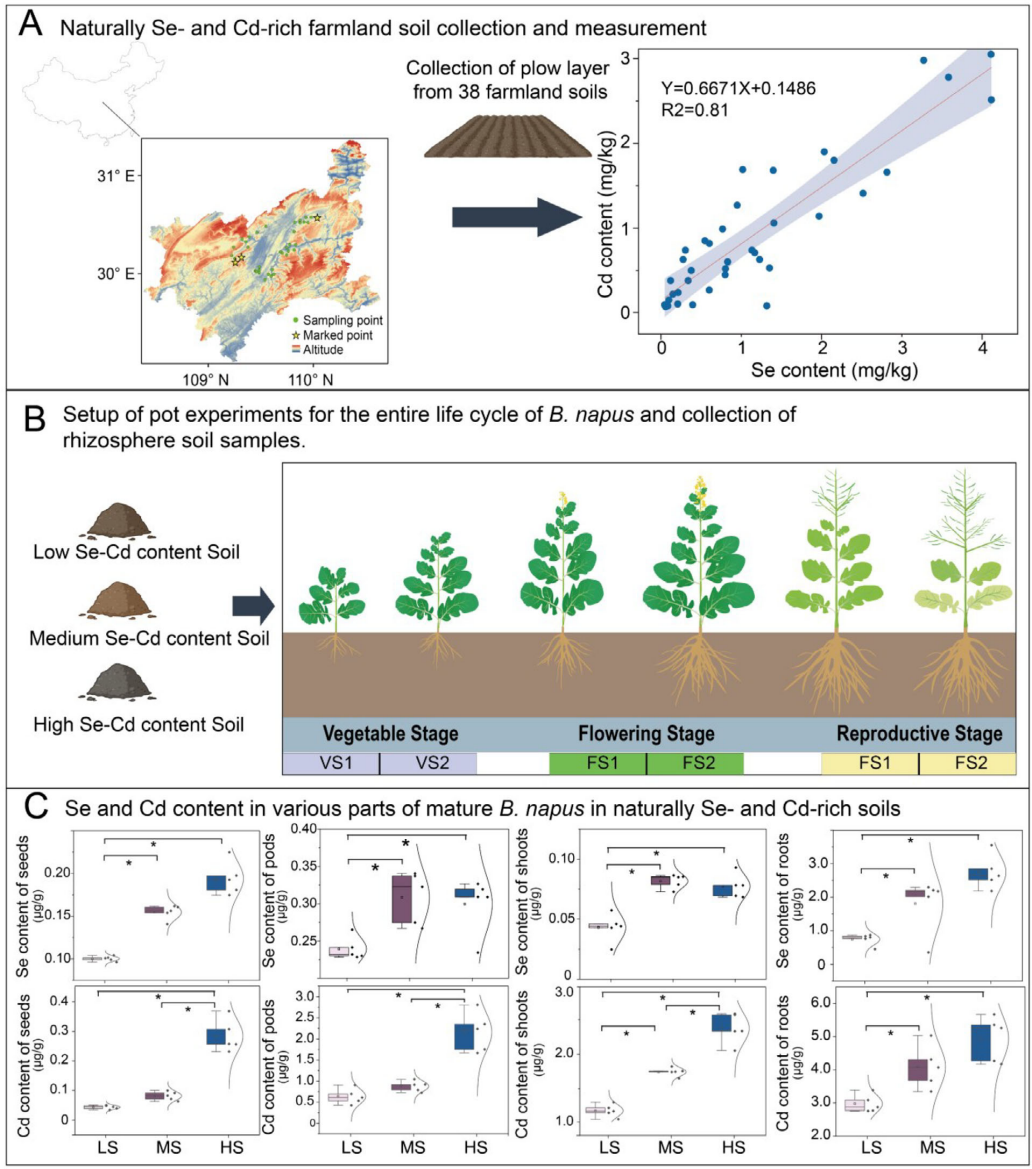
Investigation of Se and Cd coexistence in farmland and pot experiments throughout the entire growth cycle of *B. napus*. A) Schematic diagram of sampling sites for farmland soils that were naturally enriched in Se and Cd, including analysis of the co‐occurrence patterns of Se and Cd. The diagram shows 38 sampling sites, with labeled points indicating the soil sample collection locations utilized for subsequent pot experiments. The right graph illustrates the correlation between the total Se and total Cd content across the 38 sites, demonstrating a strong co‐occurrence of Se and Cd in the sampled agricultural regions. B) Schematic diagram of the entire growth cycle of *B. napus* cultivated in various agricultural soils that were naturally enriched in Se and Cd, with rhizosphere soil samples collected at six developmental stages (VS1: vegetative stage 1, VS2: vegetative stage 2, FS1: flowering stage 1, FS2: flowering stage 2, RS1: reproductive stage 1, and RS2: reproductive stage 2). C) Se and Cd concentrations in different plant parts (seeds, pods, shoots, and roots.) of mature *B. napus* grown in the three types of naturally Se‐ and Cd‐enriched soils (LS: low Se and Cd content soil, MS: medium Se and Cd content soil, HS: high Se and Cd content soil). The asterisks denote the level of significance (^*^ *p* < 0.05, ^**^ *p* < 0.01, ^***^ *p* < 0.001) among different samples based on a one‐way ANOVA test with Dunnett's post hoc analysis (for data with normal distributions and homogeneous variance) or Kruskal‐Wallis test with Dunn's post hoc analysis (for data that do not fit normal distributions or do not have homogeneous variance), n = 5.

The main objective of this study is to investigate whether and how plants regulate the dynamics of rhizosphere nutrient availability by manipulating the rhizosphere microbiome throughout their entire growth phase. We first examined the co‐occurrence of Se and Cd in the farmland in Enshi, China, which known as the “Se capital of the world” (**Figure** **1**A). Pot experiments with representative soils containing different levels of Se and Cd were set up to perform non‐destructive continuous sampling of rhizosphere soil. The rhizosphere soil of the local main crop *Brassica napus* (*B. napus*) was collected at six different stages of its growth cycle. The different chemical forms of Se and Cd in the rhizosphere were determined, and 16S rDNA gene sequencing and metagenome sequencing were performed. We assume that: 1) the dynamic changes of rhizosphere microbiome significantly influences the chemical forms of Se and Cd as plants progress through the various developmental stages; 2) there is a group of rhizosphere microbiotas that are highly related to the availability of Se and Cd and participate in the rhizosphere Se and Cd cycle; and 3) constructing a synthetic microbial consortium from this group of rhizosphere microbiotas regulate the availability of Se and Cd in soils.

## 2 Results

### 2.1 The Se and Cd Content of *B. napus* in Various Naturally Se‐ and Cd‐Rich Soils

To investigate the co‐occurrence of Se and Cd in farmland soil, we collected 38 soil samples from the cultivated layer in the Enshi region (Figure 1A; Table S1, Supporting Information). Analysis of the total Se and Cd content in these 38 soil samples revealed a strong positive correlation between soil Se and Cd levels (linear regression: Y = 0.6671X + 0.1486, R^2^ = 0.81), with an average total Se and Cd content of 1.19 and 0.94 mg kg^−1^, respectively. Among these, soils classified as Se‐rich (> 0.3 mg kg^−1^) constituted 76.32% of all the samples, and the proportion of the samples where Cd exceeded the standard (> 0.3 mg kg^−1^) was 76.92% (Figure 1A).

Furthermore, we characterized the changes in the available Se and Cd content and the associated microbiome in the rhizosphere of plants at various developmental stages (45, 90, 120, 150, 180, 210 days post‐germination) grown in naturally Se‐ and Cd‐rich farmland soils. We chose the predominant local crop, *B. napus*, and soils with three distinct levels of Se and Cd (within the concentration range of Se and Cd typically observed in Enshi farmland soils) to establish a non‐destructive, continuous rhizobox system for sampling rhizosphere soil at various stages throughout the plants’ development (Figure 1B; Figure S1, Supporting Information), as previously described.^[^ ^22^ ^]^

Next, we determined the Se and Cd content in the roots, stems, pods, and grains of mature *B. napus* grown in the three different soils (Figure 1C). The results showed a strong positive correlation between the Se and Cd content in the plants and in the corresponding soils, with the highest Se and Cd concentrations found in the roots, which were 0.78 ± 0.18, 1.89 ± 0.86, and 2.87 ± 0.53 µg g^−1^ for Se and 2.90 ± 0.27, 3.85 ± 0.60, and 5.05 ± 0.73 µg g^−1^ for Cd in the soils with low (Lsoil), medium (Msoil), and high (Hsoil) Se and Cd content, respectively. The Se and Cd content in the seeds, which is an economically crucial organ for an oil crop like *B. napus*, deserved particular attention. It was significantly correlated with the soil's Se and Cd background values (the Se content of the seeds in Lsoil, Msoil, and Hsoil was 0.11 ± 0.01, 0.17 ± 0.02, and 0.19 ± 0.02 µg g^−1^, and the Cd content was 0.04 ± 0.01, 0.08 ± 0.01, and 0.29 ± 0.05 µg g^−1^, respectively; Figure 1C). The Se content in the seeds from all three soils exceeded the threshold for Se‐rich foods (> 0.05 mg kg^−1^), but the Cd content also surpassed the national limit for food contaminants (0.08 mg kg^−1^). Notably, the Cd content in the seeds from Hsoil exceeded this standard by 262.5%.

### 2.2 The Se and Cd Content of *B. napus* in Various Naturally Se‐ and Cd‐Rich Soils

The plant‐soil feedback (PSFs) theory emphasizes that root exudates regulate plant growth by altering the soil's physicochemical environment around the roots, promoting nutrient acquisition, and recruiting specific beneficial microbial communities.^[^ ^3^, ^23^ ^]^ The absorption of soil elements by plants largely depends on their bioavailability in the rhizosphere soil.^[^ ^3^, ^4^ ^]^ The Se and Cd availability and the microbial community characteristics of rhizosphere soil at different developmental stages of *B. napus* were monitored using a rhizobox, which performs continuous sampling of rhizosphere soil in a non‐destructive manner (Figure S1, Supporting Information).

**Figure 2:**
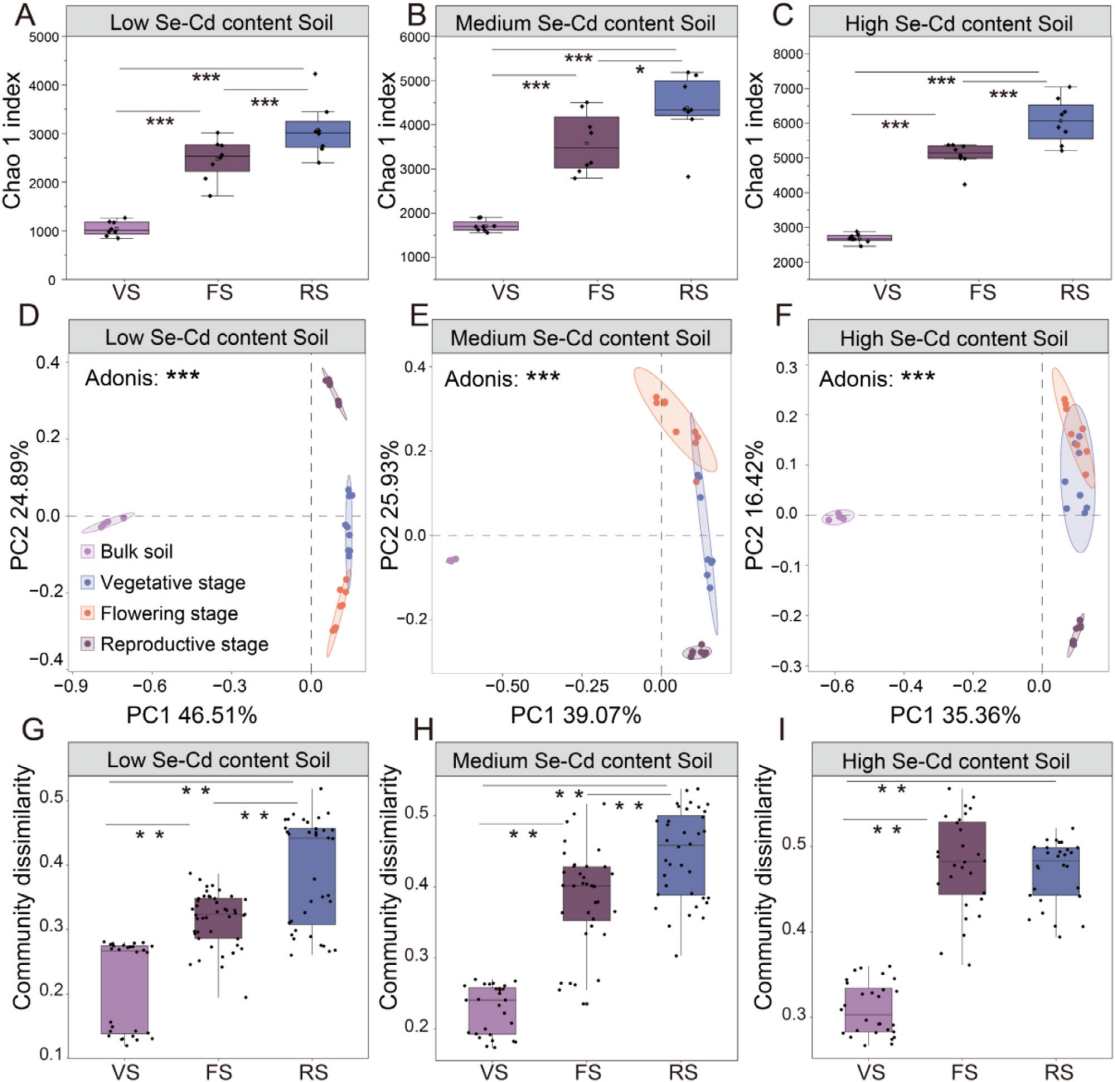
Changes in the root‐associated microbial diversity across the developmental stages of *B. napus*. A‐C) Changes in the alpha diversity of rhizosphere microbiota across the different developmental stages of *B. napus* in the three types of soil. D–F) Principal component analysis (PCA) of the rhizosphere microbial community structure in the three types of soil across the different developmental stages of the plants. Here, “Bulk soil” represents the non‐rhizosphere soil, corresponding to the first rhizosphere sample collection, indicating that the soil in the root bags was significantly influenced by the activity of *B. napus* root systems in the first rhizosphere sample collection. G–I) The community dissimilarity index based on the Bray‐Curtis distance was used to compare the differences among the microbial communities at different developmental stages of the plants in the three types of soil. The asterisks represent the level of significance (^*^ *p* < 0.05, ^**^ *p* < 0.01, and ^***^ *p* < 0.001) among different samples based on a one‐way ANOVA test with Dunnett's post hoc analysis.

As plant development progressed, the Chao1 index in the rhizosphere of the three soils significantly increased (**Figure** **2**A–C). The rhizosphere bacterial communities were also strongly influenced by the plant growth stage in the three soils (Figure 2D–F; Adonis, *P* < 0.001). The microbial communities in the bulk and rhizosphere soils were significantly separated across the first principal axis, indicating that the rhizosphere strongly affected the composition of the rhizosphere microbiome (Figure 2D–F). The rhizosphere bacterial communities of plants at different developmental stages separated along the second axis (Adonis, *P* < 0.001). The microbial communities from the early stages, notably the vegetable stage (VS) and flowering stage (FS), were relatively similar, while those from the reproductive stage (RS) were different. In addition, as the plants developed, the rhizosphere microbial communities exhibited higher community dissimilarity (Figure 2G–I).

We also examined the microbial communities from rhizosphere soils with three different Se and Cd concentrations (Figures S2 and S3, Supporting Information). The microbial communities in the three soils showed significant differences at different developmental stages of the plants (Adonis, *P* < 0.001), which was consistent with the pattern observed in the bulk soil that was not affected by plants (Figure S3, Supporting Information). Although the three soils exhibited similar community succession, with significant changes across the plant developmental stages, the communities growing at different soil Se and Cd concentrations did not become more similar (Figure S3A—D, Supporting Information).

### 2.3 Dynamic Succession of the Rhizosphere Microbiome in Soils with Different Levels of Se and Cd

**Figure 3:**
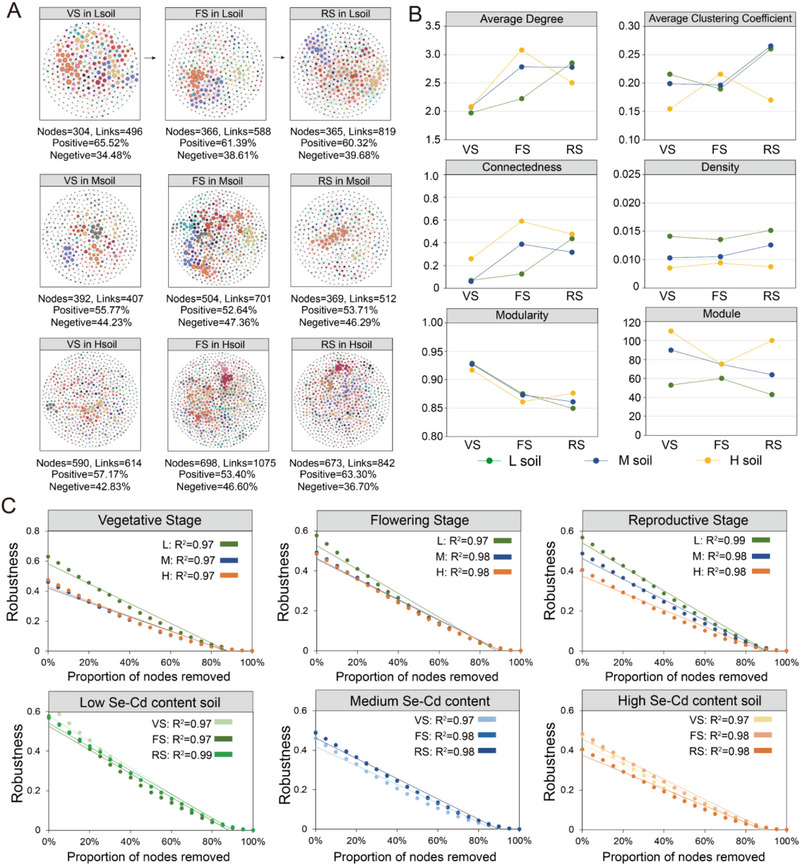
Network characteristics of root‐associated microbial communities in three types of Se‐ and Cd‐rich soils. A) Network associations and modularity. Domain‐specific molecular ecological networks were constructed based on the iNAP platform for the different stages of plant development, including the ecological networks for the vegetable stage (VS1, VS2), flowering stage (FS1, FS2), and reproductive stage (RS1, RS2). Based on the theory of random matrices, a set of thresholds for network construction was generated. To ensure comparability among different networks, a uniform threshold (cut‐off = 0.97) was used to screen for important associations among ASVs. B) Dynamic changes in the topological properties of microbial networks across different plant developmental stages in the three types of soil. C) Microbial network stability at different developmental stages and Se‐ and Cd‐rich soils, computed by calculating the average degree and network connectivity after randomly removing a certain proportion of nodes.

Dynamic rhizosphere microbial communities reflect the developmental needs of plants at different stages of growth.^[^ ^5^, ^23^ ^]^ First, we built co‐occurrence networks to detect microbiome members’ co‐occurrence patterns throughout plant development in naturally Se‐ and Cd‐rich farmland soils (**Figure** **3**). The degree distribution of nodes in all nine networks followed a power‐law distribution, with all R^2^ values greater than 0.96, which reflected the scale‐free and nonrandom features of the networks (Figure S4, Supporting Information). The results showed no significant differences in the modularity of the rhizosphere microbial networks among the soils with different levels of Se and Cd, and they showed similar decreasing trends at different developmental stages of the plants. The average degree and connectivity of the rhizosphere microbial networks in Msoil and Hsoil first increased and then decreased. Meanwhile, an increasing trend with plant development occurred in Lsoil, and the greatest differences were observed in the three soils at the flowering stage. By randomly removing nodes to simulate species extinction and calculating the network robustness after each node removal, we found that the rhizosphere microbiome in Lsoil had higher network stability (Figure 3C). Additionally, analyzing the community assembly processes across the entire plant developmental cycle in the three soils revealed that deterministic processes showed an upward trend with increasing selenium and cadmium concentrations (Figure S5, Supporting Information). These results indicated that the Se and Cd content in the soil had a strong impact on the dynamic succession of the rhizosphere microbiome of *B. napus*.

### 2.4 Core Microbiome Composition and Construction of Key Microbial Clusters Highly Correlated with Soil Available Se and Cd

**Figure 4:**
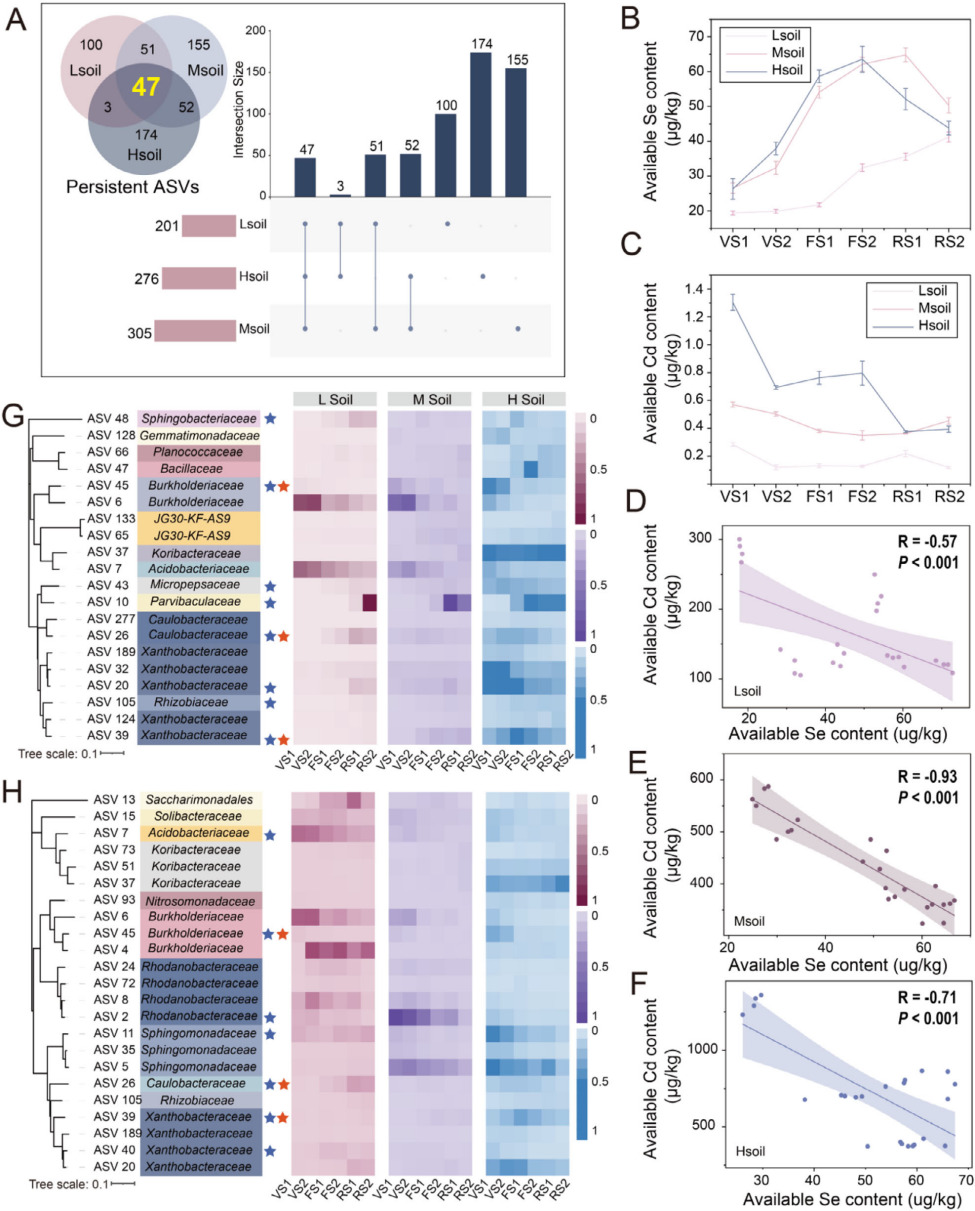
Key microorganisms related to Se and Cd available during plant development. A) Use of the abundance‐occupancy model to identify core microbes across temporal scales. A total of 201, 305, and 276 core ASVs were identified in the three types of soils, respectively, with 47 ASVs defined as core microbes in all three soils. B,C) Dynamic changes in the available Se and Cd content in the rhizosphere at different developmental stages of *B. napus*. D–F) Correlations between the available Se and Cd content in the rhizosphere among the three naturally Se‐ and Cd‐rich soils. G,H) Highly correlated ASVs identified by random forest models constructed with core microbes and the availability of Se and Cd in the rhizosphere. The listed ASVs are among the top 10 most important ASVs across the three soils. Those marked with blue stars are ASVs with high correlation in all three soils, and those with red stars are ASVs that are stably present and highly correlated with both available Cd and Se across the three soils. The heatmap on the right shows the relative abundance of these ASVs in the three rhizosphere soils at different developmental stages of *B. napus*.

Abundance‐occupancy distribution was used to explore the persistent core members of the microbiome in each of the naturally Se‐ and Cd‐rich soils. The heterogeneity of soil may induce differences in the composition of the core microbial members. We conducted separate analyses on the three types of soil, taking into consideration the heterogeneity of the core microbiome in the different Se‐ and Cd‐rich soils (Figure S6A—C, Supporting Information). A total of 201, 305, and 276 core ASVs were identified in Lsoil, Msoil, and Hsoil, respectively, with relative abundances accounting for 76.07%, 65.35%, and 47.51% of their respective total ASVs. We identified a total of 47 ASVs in common across the three Se‐ and Cd‐rich soils, consisting mainly of Proteobacteria (61.70%), Acidobacteria (21.28%), and Firmicutes (6.38%; **Figure** **4**A; Figures S6D,S7, Supporting Information). They persisted and were highly abundant in the rhizosphere of Se‐ and Cd‐rich soils. Therefore, they were defined as the core members of the rhizosphere bacterial community of *B. napus* arcoss its entire developmental period.

Next, we focused on the dynamic changes in the Se and Cd fractions in the three types of rhizosphere soil of *B. napus* (Figure S8, Supporting Information). In the soils with low Se and Cd content, available Se in the rhizosphere displayed a gradually increasing trend, while that in the roots growing in soils with moderate and high levels of Se and Cd displayed an increasing and then decreasing trend, with peaks occurring in the late flowering and early reproductive stages. The available Cd content in the three rhizosphere soils displayed a decreasing trend, with a peak at the vegetable stage 1 (Figure 4B,C). In addition, we conducted correlation analysis on available Se and Cd in the three types of soil. Interestingly, the available Se and Cd content in the rhizosphere of the three soil groups were significantly negatively correlated (*P* < 0.001; Figure 4D–F).

We've also measured the dynamics of other soil factors across different developmental stages (Figure S9, Supporting Information). Results showed that other environmental factors, like soil pH, exhibit varying correlation trends with available Se and Cd, indicating their potential influence during plant development. Available Se is significantly negatively correlated with ammonium – nitrogen and nitrate – nitrogen content but positively correlated with soil pH and organic matter. Available Cd is significantly negatively correlated with ammonium – nitrogen but positively correlated with nitrate – nitrogen and soil organic matter. Notably, available Se and Cd are significantly positively correlated here due to integrated data from all three soils, with their absolute contents mainly related to initial soil Se and Cd levels. In contrast, available Se and Cd in rhizosphere soil at different plant growth stages show a significant negative correlation. Rhizosphere microbiome strongly affects the availability of nutrients in the rhizosphere. We speculated that changes in the microbial community composition in the rhizosphere during the development of *B. napus* are involved in mediating changes in the levels of available Se and Cd in the soil.

Next, random forest machine learning models were established to regress the rhizosphere microbial relative abundances and available Se and Cd content in the three Se‐ and Cd‐rich soils at the ASV level. These models aimed to find core ASVs that are highly associated with available Se and Cd in the soil across plant development. Considering the differences in the background values of the Se and Cd content in the soils, combining the three soil groups to construct a random forest model had the potential to yield many false positive results. Therefore, we chose to construct separate random forest models for each of the three soils and performed 10‐fold cross‐validation implemented with five repeats to evaluate the potential association rankings with dynamic changes in the available Se and Cd fractions (Figure 4G,H). The top 10 ASVs were selected as the key microbiome members related to available Se or Cd in each soil. Then, the dynamic abundance characteristics of the key ASVs were used for regression fitting with the soil available Se and Cd content, which could explain 93.79% (average explanatory power in three soils) and 88.19% (average explanatory power in three soils) of the changes in the soil available Se and Cd content, respectively (Figure 4G‐H)

**Figure 5:**
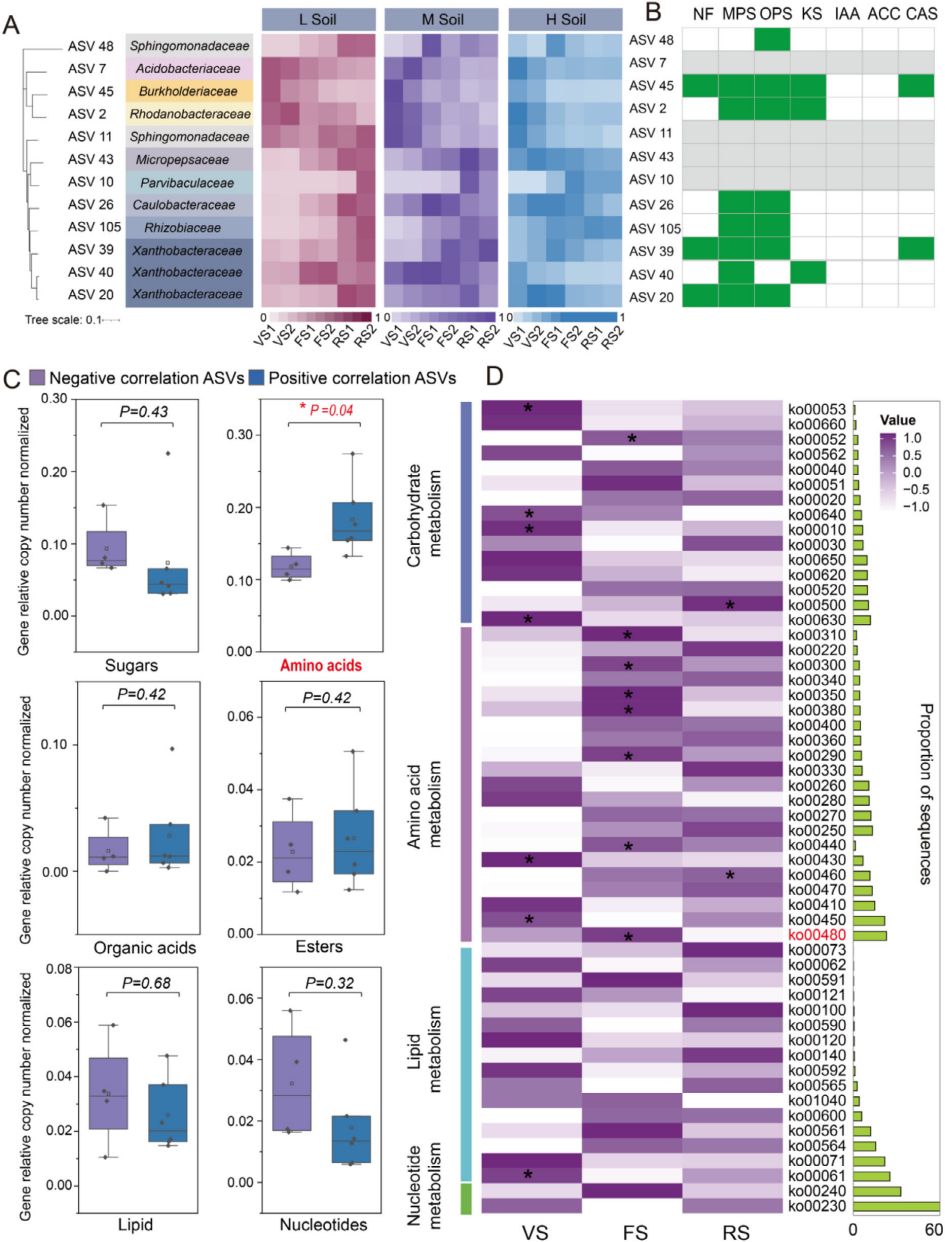
Key microbial growth‐promoting functions, genomic and metagenome analysis. A) Phylogenetic tree of key ASVs that are highly correlated with Se and Cd content in the rhizosphere and their dynamic changes in relative abundance in the rhizosphere of the three types of soil across different developmental stages of *B. napus*. B) Measurement of plant growth‐promoting functions of key ASVs. Green indicates the presence of the function, white indicates the absence of the function, and gray indicates that the microorganism was not isolated. NF indicates nitrogen – fixation potential; MPS, inorganic phosphate – solubilizing potential; OPS, organic phosphate – solubilizing potential; KS, mineral potassium (K) – solubilizing potential; IAA, ability of strains to produce indole – 3 – acetic acid; ACC, ability to produce ACC deaminase; CAS, ability to produce siderophores. C) Genomic analysis of substrate uptake capabilities conducted on members that were positively correlated with available Se and negatively correlated with available Cd, as well as members that exhibited the opposite trend. All gene frequencies were adjusted for differences in genome size, with the total number of transporters shown as the percentage of transporters per genome. In each box plot, a point denotes a single metabolic trait or a single gene. Differences in the distribution of traits between the two groups of isolates were evaluated using the Kruskal–Wallis one‐way analysis of variance, and traits with significant differences (^*^ *p *< 0.05) were identified. Positive responders: *n*  =  6, and negative responders: *n*  =  4. D) Metabolic pathway analysis based on the relative abundance of key substrates in the metagenomes at different developmental stages of the plants. The pathway marked in red (Ko00480) has the highest proportion of sequences belonging to amino acid metabolism (^*^ *p* < 0.05).

A phylogenetic tree was built for these key Se‐ and Cd‐related ASVs. The microbial members in the two clusters had a certain degree of overlap at the family level (Figure 4G,H). It was worth noting that eight stable ASVs were highly correlated with available Se and seven ASVs were highly correlated with available Cd in the three naturally Se‐ and Cd‐rich soils. Three ASVs also shared by available Se and Cd. We defined these 12 ASVs as key microbial clusters related to the bioavailability of Se and Cd in the rhizosphere (**Figure** **5**A). These ASVs were highly correlated with available Se and Cd in the plant rhizosphere. According to their positive and negative correlation with available Se content and available Cd content, these microbial members were divided into positive response clusters and negative response clusters.

### 2.5 Isolation of Se‐ and Cd‐Related Strains and Identification of Their Plant Growth‐Promoting Functions

To further verify the specific functions of the Se‐ and Cd‐related microorganisms identified by the random forest models, we isolated rhizosphere microorganisms from the three types of soil using the high‐throughput isolation and identification method.^[^ ^24^ ^]^ A total of 393 different strains were isolated, distributed among 122 genera (Figure S10, Supporting Information). Sequence alignment of these strains with key microbial cluster members related to the bioavailability of Se and Cd in the rhizosphere identified eight matching ASVs (similarity > 97%).

For these strains, we identified their plant growth‐promoting functions (including the ability to fix nitrogen, mineralize organic/inorganic P, solubilize K, and produce IAA, ACC enzymes, and siderophores). Contrary to expectations, these strains did not exhibit common plant rhizosphere growth‐promoting functions, such as IAA‐production and ACC enzyme activity, but most strains exhibited P solubilization activity (organic P mineralization and inorganic P mineralization; Figure 5B).

In addition, we determined the Se reduction and Cd adsorption capabilities of these isolated strains, which indirectly reflects their ability to activate or immobilize environmental Se and Cd. The results indicated that these strains possess strong Se reduction capabilities (24.09–88.48%) and relatively strong Cd adsorption capabilities (10.78–33.03%; Figure S11, Supporting Information). We also set up a co‐cultivation treatment with both Se and Cd. Interestingly, we found that, compared to cultivating in environments with either Se or Cd alone, the strains demonstrated significantly enhanced transformation capabilities for both Se and Cd under co‐existing conditions. This suggests that the transformation of environmental Se and Cd by these strains may not be independent but could potentially involve a shared metabolic regulatory pathway.

### 2.6 Substrate Preference of Available Se‐ and Cd‐Related Microbes Based on Genome and Metagenome Mining

Plants employ different strategies when releasing root exudates at different developmental stages to regulate the rhizosphere microbiome and meet the plants’ functional needs at each stage.^[^ ^9^, ^25^ ^]^ Based on the persistent presence of key microbial clusters that are highly correlated with soil available Se and Cd and their highly dynamic characteristics during plant development, we speculated that these positive cluster members may have similar substrate preferences for root exudates. These preferences, in turn, drive the enhancement of certain metabolic pathways and promote the activation of Se and Cd in the rhizosphere soil. To verify this hypothesis, we conducted a genomic analysis on key cluster members related to Se and Cd (including NCBI‐reported genomes and MAGs (metagenome‐assembled‐genomes) from our experiment that are based on metagenomic binning results with a completeness of 100% and contamination level below 1.5%).

We analyzed the genomic characteristics of these microbial members, and the Hidden Markov Model search targeting TransportDB was used on the genomes to predict transporter proteins involved in substrate uptake (Table S3, Supporting Information). The results indicated that the genomes of positive cluster members had higher copy numbers of amino acid transporter genes compared to the genomes of negative cluster members. Meanwhile, the results also suggested that the negatively correlated ASVs have a higher likelihood of having sugar and nucleic acid transporters, but these trends were not statistically significant (Figure 5C). In addition, we analyzed metagenomic data from different developmental stages of the plants by annotating and comparing the KEGG metabolic functions from the vegetative, flowering, and reproduction stages (Figure 5D). A higher number of genes belonged to the KEGG pathway related to amino acid metabolism at the flowering and reproductive stages, while the genes involved in certain pathways related to sugar and nucleic acid metabolism were more abundant at the vegetable stage (Figure 5D). These results suggested that plants may drive the enrichment of key microbial clusters that are highly correlated with soil available Se and Cd in the rhizosphere by secreting more amino acids during the flowering and reproduction stages. Among all the amino acid metabolic pathways and differential amino acid metabolic pathways, glutathione metabolism had the highest proportion of represented sequences. This gene abundance peaked during the flowering stage, leading to the speculation that the glutathione metabolic pathway significantly regulated the changes in available Se and Cd in the soil as the plant's development progressed.

Next, we constructed a gene set related to Se and Cd from the soil metagenome. PCA analysis conducted using the NR database based on this gene set yielded results similar to the PCA analysis using the NR gene set. Significant differences were observed at different developmental stages, indicating that the microbial communities related to Se and Cd underwent significant changes at various developmental stages of the plants (Figure S12, Supporting Information). In addition, we analyzed the abundance changes of these genes at different developmental stages by performing a functional analysis of the differentially abundant genes using KEGG. The results indicated that the top three genes related to soil Se cycling at the various plant developmental stages showed a mean proportional gene abundance that was higher at the flowering stage than at the reproductive stage and higher at the vegetative stage. This trend was the same as what was observed in the available Se content in the soil (Figure S13, Supporting Information). Among them, the gene with the highest mean proportion, *LysC*, is involved in encoding aspartate kinase and plays a role in the metabolism of glycine, serine, threonine, cysteine, and methionine (Figure S12C, Supporting Information). Among the four significantly differentially abundant genes related to microbial Cd, the *merP*, *maeB*, and *czcC* gene showed significantly higher expression in the flowering stage or reproductive stage compared to the vegetable stage. The *Zrt* and *cusC* gene, related to the absorption and transport of Cd ions, showed significantly higher expression during the early stages of plant growth compared to the flowering and reproductive stages (Figure S13, Supporting Information). This may be related to the decrease in the concentration of available Cd ions in the rhizosphere environment as the developmental stage progressed.

### 2.7 Glutathione Metabolism‐Related Amino Acids‐Enhanced Synthetic Microbial Community (SynCom) Promotes Plant Se Enrichment and Cd Reduction

Metagenomics analysis revealed that genes related to glutathione metabolism participate in amino acid metabolic pathways and exhibit significant differences across various developmental stages of *B. napus*. Consequently, it was hypothesized that plants secrete amino acids related to this pathway at different stages of development, which, in turn, recruit microorganisms with preferences for such amino acid substrates and promote glutathione metabolism. We constructed a synthetic metabolic substance comprising the core amino acids of glutathione‐cysteine, glycine, and glutamate, which are essential for the metabolic pathway of glutathione.

**Figure 6:**
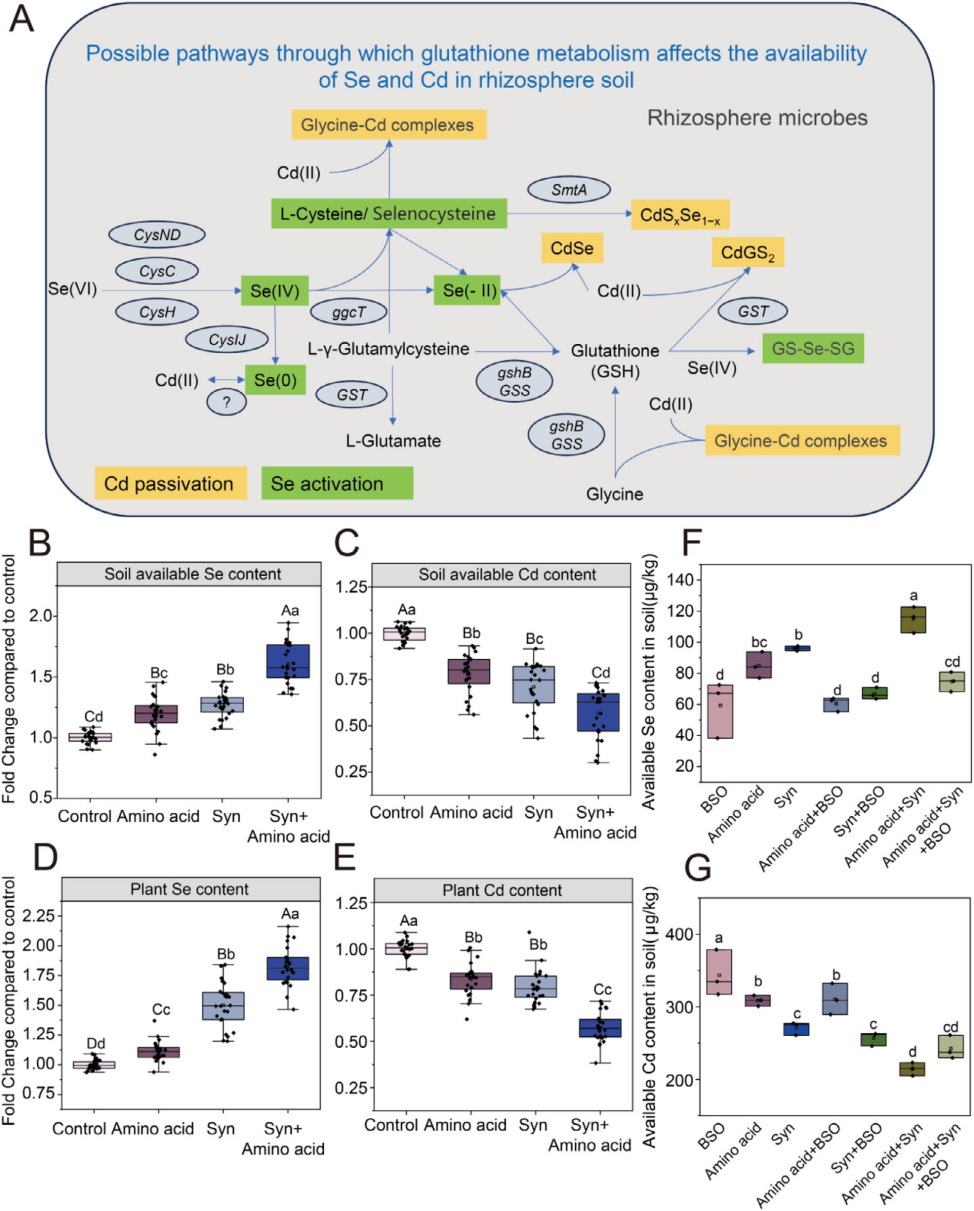
The process of Se‐enrichment and Cd‐reduction in soil affected by the glutathione metabolic pathway, and the verification experiments of pot culture and soil cultivation. A) Flowchart showing the possible pathways through which glutathione metabolism affects the availability of Se and Cd in rhizosphere soil throughout the plants’ development. The green labels indicate the Se activation pathway, the yellow labels indicate the Cd immobilization pathway, and the italicized labels are the names of the genes that regulate this process B,C) Soil incubation experiments to verify that the SynCom and key glutathione metabolic pathway amino acids increase the available Se content and decrease the available Cd content in the soil. To verify the widely applicable and stable effects of the SynCom and key glutathione metabolic pathway amino acids on Se enrichment and Cd reduction, verification experiments were conducted on eight naturally Se‐ and Cd‐enriched soils. Among them, “Control” represents the treatment amended with ultrapure sterile water, “Amino acid” represents the treatment amended with the three key glutathione metabolic pathway amino acids, “Syn” represents the treatment inoculated with the SynCom, and “Syn + Amino acid” represents the treatment co‐inoculated with the SynCom and the three key glutathione metabolic pathway amino acids. Specific data can be found in Figures S14,S15 (Supporting Information). The x‐axis represents the difference in the levels of available Se and Cd in the soil compared to the control treatment and expressed as a multiple of the difference. Different letters indicate significant differences in the plant growth phenotypes in the different treatments at *p* < 0.05 (lowercase) or *p* < 0.01 (uppercase), which were tested using a one‐way ANOVA. D‐E) Potted experiments to verify the effects of the SynCom and the glutathione key glutathione metabolic pathway amino acids on the plant Se uptake and Cd absorption. F‐H) Experiments to verify the activation of Se and immobilization of Cd by the glutathione metabolic pathways. In these experiments, BSO refers to the treatment with 100 µM buthionine sulfoximine, an inhibitor of the glutathione metabolic pathway.

Next, six strains (ASV20, ASV26, ASV39, ASV40, ASV48 and ASV105) were used to construct a synthetic microbial community (SynCom) with Se – enrichment and Cd – reduction potential. The strains in this community were isolated and identified through high – throughput methods. They showed a strong positive correlation with available Se or a strong negative correlation with available Cd during plant development. Moreover, they are stable members of the persistent microbiota that can stably colonize the plant rhizosphere across different developmental stages and these strains did not show mutual growth inhibition (Figure S14, Supporting Information). We performed mutual growth interaction assays among synthetic community members and the results demonstrated the absence of mutual inhibitory effects among strains, ensuring the synthetic community's stability. To verify whether the artificially constructed SynCom and key glutathione metabolic pathway amino acids could increase the levels of available Se in naturally Se‐Cd‐rich soils, reduce the available Cd content, and increase the Se content in plants while reducing the Cd content, we conducted soil culture experiments and pot experiments using eight types of naturally Se‐ and Cd‐rich soils (Figures S15,S16, Supporting Information). The results showed that, compared with the control, adding key glutathione metabolic pathway amino acids alone significantly increased the available Se content by 19.60% and significantly decreased the available Cd content by 21.94% (**Figure** **6**). Inoculating with the SynCom alone significantly increased the available Se content by 26.96% and reduced the available Cd content by 28.95% (Figure 6B,C). Co‐inoculating with key glutathione metabolic pathway amino acids and the SynCom significantly increased the available Se content by 60.57% and significantly decreased the available Cd content by 43.21% (Figure 6B,C). The results from the pot experiments were similar to those of the soil culture experiments. Adding key glutathione metabolic pathway amino acids or inoculating with the SynCom alone significantly increased the Se content by 11.41% and 50.07%, respectively, and significantly reduced the Cd content by 16.39% and 21.45%, respectively (Figure 6D,E). Co‐inoculating with key glutathione metabolic pathway amino acids and the SynCom significantly increased the plants’ Se content by 82.66% and significantly reduced their available Cd content by 42.49% (Figure 6D‐E). These results indicate that the constructed SynCom and synthetic key glutathione metabolic pathway amino acids can significantly increase the Se content in plants and reduce Cd absorption through soil nutrients availability. Moreover, co‐inoculating with both substances can achieve an even more positive effect, suggesting that combining key microbes with the glutathione metabolic pathway is a highly promising approach to reduce plant Cd absorption and enhance Se absorption, with broad application prospects in farmlands where Se and Cd co‐occur.

Additionally, we set a 14 – day pot experiment with three naturally Se‐Cd‐rich soils and a 7 – day plant agar culture experiment to assess the impact of microbes and amino acids on plant growth (Figure S17, Supporting Information). The results showed that in different soils, applying key amino acids related to the glutathione metabolic pathway and inoculating synthetic microbial communities significantly increased the fresh and dry weight of *B. napus*. Notably, in Lsoil, where no initial fertilizer was added, the older leaves of oilseed rape showed clear nitrogen – deficiency symptoms. However, adding amino acids and synthetic microbial communities alleviated this situation. Similarly, the agar culture results demonstrated that these applications significantly enhanced oilseed rape's root length. These findings indicate that applying amino acids related to the glutathione metabolic pathway and inoculating synthetic microbial communities not only affect the availability of selenium and cadmium in the rhizosphere but also significantly improve the growth of *B. napus*.

Our study highlights the significant role of microbial glutathione metabolism in regulating the availability of Se and Cd in the rhizosphere during plant development. To verify this hypothesis, soil cultivation experiments amended with L‐buthionine sulfoximine (BSO), an inhibitor of the glutathione metabolic pathway, were conducted. The results indicated that inhibiting glutathione metabolism significantly reduced the available Se content in the rhizosphere by 35.03% when glutathione key glutathione metabolic pathway amino acids and the SynCom were added (Figure 6F,G). In contrast, the impact of BSO on available Cd in the rhizosphere was relatively small, increasing it by 13.55% (Figure 6F,G). This suggests that inhibiting the glutathione metabolism process in the soil can have a negative impact on the bioavailability of Se and the immobilization of Cd in the soil.

In addition, RT – qPCR was used to demonstrate how microbe may affect Se and Cd transformation via metabolic pathways. ASV26 (*Phenylobacterium zucineum* HLK1) from the synthetic community was used as the test strain because it had the highest relative abundance among strains in the positive group that showed a strong positive correlation with available selenium and a strong negative correlation with available cadmium (Figures S18,S19, Supporting Information). The result showed that, compared to the control treatment, the presence of Se and Cd in the environment upregulates the expression of microbial genes related to glutathione metabolism. Specifically, the expression of *gshA* and *gshB*, which encode the key rate – limiting enzyme γ – glutamylcysteine synthetase and its regulatory subunit, increased by 6.76 – and 3.07 – fold, respectively. The expression of *gsiA* and *gsiB*, involved in glutathione transport, rose by 5.13 – and 5.82 – fold, respectively. Meanwhile, the expression of *TcyC*, *gltL*, and *ArtT*, which are involved in the transport of cysteine, glutamate, and arginine, increased by 1.38 ‐, 2.00 ‐, and 1.95 – fold, respectively.

In addition, the addition of key amino acids for glutathione synthesis significantly increased the expression of genes related to glutathione synthesis and metabolism in microbes. Conversely, adding the glutathione synthesis inhibitor BSO led to a marked decrease in the expression of genes encoding the rate – limiting enzyme for glutathione synthesis, along with a general decline in glutathione metabolism – related gene expression. These findings are consistent with the changes in soil Se and Cd availability observed in soil‐culture experiments, indicating that the glutathione metabolic pathway significantly contributes to the Se – enrichment and Cd – reduction effects of synthetic communities and key glutathione – related amino acids in natural Se – and Cd – associated soils.

## 3 Discussion

Inoculating with PGPB is an effective way to improve plant nutrient absorption and has great potential for the sustainable development of agricultural ecosystems.^[^ ^4^, ^26^ ^]^ However, environmental dependence, host selectivity, and persistent colonization by beneficial strains are challenges that inoculated microbial agents need to overcome.^[^ ^27^ ^]^ We screened the core microbiome that persistently exists in the rhizosphere of *B. napus* grown in different naturally Se‐ and Cd‐rich soils throughout the entire growth cycle of the plants and constructed a SynCom based on its strong correlation with the available Se and Cd content and the growth promoting function of its strains (Figures 5 and 6). We found that the SynCom could promote Se absorption by the plant and inhibit Cd absorption by increasing the available Se content and reducing the available Cd content in the soil. In addition, this SynCom could stably function in various naturally Se‐ and Cd‐rich soils (Figure 6). Our research highlights the important role of core microbiota that are highly conserved and persistent in the host rhizosphere across a broad range of environmental conditions.

Rhizosphere microbiome is recruited by the roots to meet the plant's functional needs, and this phenomenon has been extensively studied and widely recognized.^[^ ^7^, ^28^ ^]^ Many studies have confirmed that the dynamic changes in the root exudates create different rhizosphere environments and microbial substrate preferences and drive the succession of various rhizosphere microbiomes.^[^ ^3^, ^5^, ^9^ ^]^ In this study, the genomics analysis results revealed that the core microbial cluster members that were highly positively correlated with available Cd in the soil and highly negatively correlated with available Cd across different developmental stages of *B. napus* had higher amino acid substrate uptake ability compared to the core microbial cluster members that followed the opposite trend (Figure 5). The metagenomics analysis also found that the rhizosphere soil in the highly enriched stage of positive group members had higher numbers of enriched genes related to amino acid metabolism pathways than negative group members (Figure 5). Therefore, amino acid metabolism by the rhizosphere microbiome may be a pivotal reason for changes in the Se and Cd availability in the soil. Our findings are supported by a recent study by Feng et al., in which they collected and measured the composition of root exudates from plants at different developmental stages and found that plants secrete more amino acids while transitioning from the vegetative stage to reproductive stage.^[^ ^29^ ^]^

The soil microbiome could catalyze a series of transformations of the Se fractions, including reduction, oxidation, methylation, and demethylation, and activate insoluble fractions of Se in the soil during these processes.^[^ ^16^, ^17^, ^20^ ^]^ Se(IV) and Se(VI) were previously thought to be the primary Se compounds that are absorbed by plants, while the bioavailability of Se(0) and Se(‐II) were relatively low.^[^ ^30^ ^]^ However, recent studies have reported that many PGPB in the rhizosphere that promote Se absorption are highly capable of reducing Se(IV) and Se(VI) in the soil to Se(0) and organic Se(‐II), such as selenocysteine.^[^ ^4^, ^31^ ^]^ This emphasizes that the pathways through which plants absorb Se‐containing amino acids are also worthy of attention. Our results showed that the SynCom, consisting of the core rhizosphere microbiota with a high ability for amino acid metabolism, combined with amino acids related to the glutathione metabolic pathway, effectively improved the soil bioavailable Se levels and plant Se absorption. This phenomenon was also stable in different naturally Se‐ and Cd‐rich soils. A recent study found that most plants are more likely to absorb organic Se, such as SeMet, and transfer and volatilize it at much higher rates than inorganic forms of Se.^[^ ^32^ ^]^ In our other study, we also found that selenocysteine produced by rhizosphere microbial metabolism was important in promoting plant Se absorption.^[^ ^4c^ ^]^

Amending the soil with key glutathione metabolic pathway amino acids metabolic substances enhanced the glutathione metabolic pathway activity. Numerous studies have uncovered the beneficial role of the glutathione metabolic pathway in soil Se reduction, with the synthesis of elemental Se and Se(‐II) within cells primarily relying on the glutathione redox system. Se(IV) and Se(VI) spontaneously react with reduced glutathione (GSH), forming GS‐Se‐SG and the oxidized form of glutathione (GSSG), ultimately transforming into Se(‐II) and elemental Se. H_2_Se is the main intermediate metabolic product in the synthesis of various forms of Se within microbial cells, eventually leading to the formation of various organic Se compounds, including selenoamino acids.^[^ ^4^, ^32^, ^33^ ^]^ Therefore, it is speculated that the increase in soil available Se observed in the validation experiments is mainly due to the production of certain selenoamino acids, such as selenocysteine. Research on Cd sequestration by the glutathione metabolic pathway has primarily focused on plants, which can reduce the absorption of Cd by acting as antioxidants and metal chelators.^[^ ^34^ ^]^ Many studies have confirmed that Cd can bind to the side chains of amino acids to form complexes, thereby reducing the environmental availability of Cd. Interestingly, some studies have shown that certain bacteria, in the presence of Cd ions, reduce selenate to form metallic CdSe‐NPs precipitates.^[^ ^35^ ^]^ It is noteworthy that the glutathione metabolic pathway is essential for the biosynthesis of CdSe in microorganisms.^[^ ^35^, ^36^ ^]^ Thus, we speculated that the glutathione metabolic pathway may actively participate in the increase of effective Se content and the reduction of effective Cd content in the soil, which is also the main reason for the significant negative correlation between effective Se and Cd content observed in the three types of soil.

## 4 Experimental Section

### Soil Preparation

Se‐ and Cd‐rich paragenesis farmland soils were collected, and the sampling points are shown in (Figure 1A). Three soils with different levels of Se and Cd were selected and denoted as Lsoil, Msoil, and Hsoil. Based on the background of this study, the coexistence of Se and Cd as an existing objective phenomenon exhibits highly regional characteristics. The combined impact of the high Se and Cd content in the soil of the parent material over a long‐time scale is far larger than that of the short‐term artificial addition of Se and Cd compounds. Therefore, different concentrations of Se and Cd were not artificially added to standard, non‐enriched soil but chose three representative soils that were naturally enriched, albeit with small differences in other physical and chemical indicators aside from Se and Cd. These soils represented the different levels of Se and Cd coexisting in farmland soils (Table S1, Supporting Information).

### Experimental Design, Plant Growth, and Sample Collection

*B. napus* was selected as the test plant because it is the largest oilseed crop in China and one of the main crops in Enshi. This prefecture is also known as the “world capital of Se”. To track the dynamic changes in the microbial community structure, functional genes, and soil nutrients in the rhizosphere soil during different stages of plant growth, a mesocosm system (“rhizobox”) was used to repeatedly collect rhizosphere soil from individual plants without damaging the plant root system.^[^ ^22^ ^]^ The schematic diagram and further explanations of the rhizobox are shown in the supporting information (Figure S1, Supporting Information). Fertilizers were applied according to the previous studies and local practices.^[^ ^4c^ ^]^

*B. napus* seeds (*Brassica napus*) were surface‐sterilized in 3% NaClO (v/v) for 5 min, rinsed four times with sterile distilled water, and germinated in the dark for 2 days. *B. napus* seedlings with similar sizes were gently planted in the root compartment of each rhizobox. There were five biological replicates per group of samples.

To collect rhizosphere soil in a nondestructive manner, three of the middle‐layer nylon bags were randomly collected at each sampling time, removing 3 centimeters of soil from the top and bottom of the nylon bag while retaining the soil in the middle area. Then, samples in each pot were pooled into one composite sample and stored at ‐80 °C until further processing. The pot experiment was conducted in October 2022. The samples were collected from 15 individual plants at the vegetative stage 1 (VS1; 45 dap), vegetative stage 2 (VS2; 90 dap), flowering stage 1 (FS1; 120 dap), flowering stage 2 (RS2; 150 dap), reproductive stage 1 (RS1; 180 dap), and reproductive stage 2 (RS2; 210 dap). The growth state of the *B. napus* roots in the root compartment was checked routinely to ensure that the root compartment was densely rooted at 45 dap and onward. The microbiome in the outer layer was compared with that of the soil in the nylon bag to evaluate whether the nylon bag soil was significantly affected by root exudates. After the final soil sample collection, plant roots, stems, pods, and seeds were collected.

### Defining Core Rhizosphere Microbiota and Available Se/Cd‐Related Microbiota Cluster Member

Rhizosphere microbiota that stably colonize the rhizosphere throughout a plant's life cycle are defined as core microbiome. The abundance occupancy distribution explores the frequency and quantity of microorganisms present in different time series and sorts them based on their frequency of occurrence to determine core persistent microbiome.^[^ ^37^ ^]^

In order to explore the potential correlation between the differences in available Se/Cd content in the rhizosphere of plants at different developmental stages and the corresponding microbial community members, a random forest machine learning model algorithm (R package: randomForest) was used to regress the in situ rhizosphere soil microbial abundance and available Se and Cd content of rhizosphere microbiome in three Se‐Cd soils at the ASV level, in order to establish a model that associates root microbial community members with soil available Se and available Cd.^[^ ^9b^ ^]^ The number of marker taxa were identified using 10‐fold cross‐validation implemented with the rfcv() function in the R package “randomForest” with five repeats. Although available Se and Cd levels in plant developmental stages vary dynamically, initial soil Se and Cd levels dominate their changes. Combining three soils would cause false positives in random forest models, so they were built separately for each soil. In the random forest model, the top 10 microbial members ranked by importance are defined as available Se/Cd related microbiota cluster key members for subsequent analysis.

### High‐Throughput Cultivation and Identification of Bacteria from the Rhizosphere Soil Microbiota

Isolation and identification of rhizosphere microbiota in mature *B.napus* from Lsoil, Msoil and Hsoil using the protocol proposed by Zhang et al (2021) with minor modifications(Figure S2, Table S4, Supporting Information).^[^ ^24^ ^]^ Briefly, the rhizosphere suspensions of three Se‐Cd soil were diluted to an optimal dilution such that 30% of the wells showed bacterial growth and amplify bacterial 16S rDNA genes in the cultivated bacteria via two‐sided barcode PCR. Cultured bacterial sequences were blast to the ASVs in corresponding rhizosphere bacteria and those showed > 97% gene identity were considered as the same bacteria. Multiple sequence alignment was performed using MEGA11 software, and the phylogenetic tree was constructed using the neighbor‐joining method with a supporting bootstrap value of 1000.^[^ ^38^ ^]^

### Analysis of Genomic Features of Positive Responders and Negative Responders

To identify differences in the substrate preference drive patterns among microbiota with different response modes, genome sequences were screened for specific traits related to substrate fitness in the rhizosphere.^[^ ^9a^ ^]^ For substrate uptake, transporters in the genomes were predicted using a Hidden Markov Model search against TransportDB.^[^ ^39^ ^]^

### Microbial Informatics Analysis

The upstream analysis progress of the amplicon sequence was processed using QIIME2 v2021.8.^[^ ^40^ ^]^ Using the DADA2 workflow in QIIME2, barcodes, PCR primers, and low‐quality sequences were trimmed, and the remaining reads were subsequently denoised and merged to generate the amplicon sequence variants (ASV) feature table. A taxonomy native Bayes classifier trained on the V3‐V4 region (338F and 806R) of the reference 16S sequence (SILVA release 138) was used to assign taxonomic identities to the representative sequences. Only ASV that were at least annotated to the phylum level were retained. Features identified as chloroplast or mitochondrial were removed. Remove low‐abundance species and standardize the final sample to the minimum sequencing depth for downstream analysis.

Shannon index of microbial alpha diversity in each sample was performed by usearch‐alpha_div (V10, http://www.drive5.com/usearch/). PCoA analysis and Bray‐Curtis distance calculation were performed by R “phyloseq” package (version 1.34.0) based on the relative abundance of ASV and using the “adonis” function of the “vegan” package in R, conduct a permutation multivariate analysis of variance to test the significance of various influencing factors on differences in bacterial communities.

To systematically study the dynamic changes of rhizosphere microbial networks driven by plant developmental stages in different Se/Cd content natural Se‐Cd rich soil, domain specific molecular ecological networks were constructed based on the iNAP platform for different developmental stages, including vegetable stage ecological networks (VS1, VS2), flowering stage ecological networks (FS1, FS2), and reproductive stage ecological networks (RS1, RS2).^[^ ^41^ ^]^ For each group of samples, ASVs present in more than 50% of the samples were selected for analysis of their ASV composition data, and Pearson correlation coefficient matrices were obtained after central logarithmic ratio transformation. Then, based on the theory of random matrices, a set of thresholds for network construction was generated. To ensure comparability between different networks, a uniform threshold (Cut off = 0.97) was used to screen for important associations between ASVs. Visualize the network using Gephi (0.9.5). Evaluation of network stability at different levels of Se‐Cd content and plant development stage nodes based on random removal of nodes from networks.

Clade‐ and community‐level assembly processes were estimated using a phylogenetic bin‐based null modelling approach, implemented in the “iCAMP” (1.5.12) R package.^[^ ^41^ ^]^ All sequence data have been submitted to the National Center for Biotechnology Information (NCBI) Sequence Read Archive under Bioproject PRJNA1193780.

### SynCom Construct and Soil Culture Microcosm and Pot Experiment Setup

Given the persistent presence of different Se and Cd levels in the rhizosphere soils of *B. napus*, various plant growth‐promoting functions, a positive correlation with available Se, and a negative correlation with available Cd, SynCom consisting of six strains (ASV 20, ASV 26, ASV 39, ASV 40, ASV 48, and ASV 105) were constructed. The strains were separately activated in TSB liquid medium for 48 h, and the optical density was adjusted to OD_600nm_ = 1. Equal volumes of each strain were pooled to generate a working stock of the SynCom. For the treatments involving the SynCom, dry soil was inoculated with a synthetic bacterial suspension to a final concentration in the soil of 1 × 10^7^ CFU·g^−1^.

An amino acid mixture containing glutamine, cysteine, and glycine was assembled. For the treatment involving the “Amino group,” each amino acid was added to the culture bottle or pot to a final concentration of 100 µM.

In order to simulate the effects of amino acid release by plants into the rhizosphere and of SynCom amendments on the soil available Se and Cd and plant Se and Cd content, four treatments: “Syn + Amino acid” (added SynCom and simulated root‐exudated key glutathione metabolic pathway amino acids), “Amino acid” (added simulated root‐exudated key glutathione metabolic pathway amino acids and sterilized SynCom), “DB” (added sterilized SynCom), “DB + Amino acid” (added sterilized SynCom and simulated root‐exudated key glutathione metabolic pathway amino acids) were set up.

To further evaluate the effects of SynCom and key glutathione metabolic pathway amino acids on a wider variety of Se‐ and Cd‐rich soils, eight different naturally Cd‐contaminated Se‐rich soils were selected for soil microcosm experiments. These experiments were conducted in 100 mL sterile culture bottles, each containing 50 g of sieved and homogenized soil. The culture bottles were sealed with a breathable sealing film and incubated under germ‐free conditions. There were 4 biological replicates per group of samples.

The pot experiments were conducted on six naturally Se‐ and Cd‐rich farmland soils, with *B. napus* as the experimental plant. Each pot contained 300 g of soil and was seeded according to the pot experiment method described earlier in this study. After 14 days of germination, the treatments were set up similar to the soil microcosm experiment. Plants were harvested after 45 days, and each treatment had five independent replicates. The pot experiments and the soil microcosm experiments were incubated under germ‐free conditions with a 16 h light cycle, 26/22 °C average day/night temperature, and relative humidity of 70%. At harvest, the root and shoot tissues were sampled. The available Se and Cd content in the soils and the Se and Cd content in the plants were determined according to the procedure described above.

### Statistical Analysis

Experimental data analysis was conducted using SPSS (version 25, IBM, USA), Origin 2021 and R‐4.2.2 and is presented as mean ± standard error (SE). Significant differences in some parameters between the different treatments were analysed using One‐way ANOVA in conjunction with Dunn‐Sidak's comparison (*P* < 0.05). A specific comparison method is shown in the figure legends. Origin 2021 was used to draw charts.

## Conflict of Interest

The authors declare no conflict of interest.

## Author Contributions

Z.L., and X.H.Z. conceived and designed this research; Z.L., H.Z., W.J.L., J.D.S., H.Z., Y.W., Y.N.T., H.X.W., C.C.D., W.Q.Q., Y.H.Z., G.Y.Y., Y.H.Z., Z.Y.L., and N.Y.Z. performed experiments; Z.L. analyzed data; Z.L., and X.H.Z. wrote the paper; C.X.H., and X.H.Z. oversaw the entire study. All authors read and approved the manuscript.

## Supporting information



Supporting Information

Supporting Information

Supporting Information

## Data Availability

The data that support the findings of this study are available from the corresponding author upon reasonable request.

## References

[1] a) R. L. Berendsen , C. M. Pieterse , P. A. Bakker , Trends Plant Sci. 2012, 17, 478; 22564542 10.1016/j.tplants.2012.04.001

[2] N. Ling , T. Wang , Y. Kuzyakov , Nat. Commun. 2022, 13, 836. 35149704 10.1038/s41467-022-28448-9 PMC8837802

[3] a) M. Zhao , J. Zhao , J. Yuan , L. Hale , T. Wen , Q. Huang , J. M. Vivanco , J. Zhou , G. A. Kowalchuk , Q. Shen , Plant Cell Environ 2021, 44, 613; 33103781 10.1111/pce.13928

[4] a) J. Liu , W. Xu , Q. Zhang , W. Liao , L. Li , S. Chen , J. Yang , Z. Wang , F. Xu , Plant Commun 2024, 5, 100930; 38685708 10.1016/j.xplc.2024.100930 PMC11369732

[5] M. Wang , A.‐H. Ge , X. Ma , X. Wang , Q. Xie , L. Wang , X. Song , M. Jiang , W. Yang , J. D. Murray , Nat. Commun. 2024, 15, 1668. 38395981 10.1038/s41467-024-45925-5 PMC10891064

[6] X. Sun , C. Jiang , Y. Guo , C. Li , W. Zhao , F. Nie , Q. Liu , J. Hazard. Mater. 2024, 473, 134587. 38772107 10.1016/j.jhazmat.2024.134587

[7] a) Y. Zheng , X. Cao , Y. Zhou , S. Ma , Y. Wang , Z. Li , D. Zhao , Y. Yang , H. Zhang , C. Meng , Nat. Commun. 2024, 15, 3520; 38664402 10.1038/s41467-024-47773-9 PMC11045775

[8] a) N. Stopnisek , A. Shade , ISME J 2021, 15, 2708; 33772106 10.1038/s41396-021-00955-5 PMC8397763

[9] a) K. Zhalnina , K. B. Louie , Z. Hao , N. Mansoori , U. N. Da Rocha , S. Shi , H. Cho , U. Karaoz , D. Loqué , B. P. Bowen , Nat. Microbiol. 2018, 3, 470; 29556109 10.1038/s41564-018-0129-3

[10] F. Wang , H. Zhang , H. Liu , C. Wu , Y. Wan , L. Zhu , J. Yang , P. Cai , J. Chen , T. Ge , Combating wheat yellow mosaic virus through microbial interactions and hormone pathway modulations, Vol. 12, Microbiome, Berlin 2024, p. 200. 10.1186/s40168-024-01911-z PMC11481568 39407339

[11] a) C. Chen , M. Wang , J. Zhu , Y. Tang , H. Zhang , Q. Zhao , M. Jing , Y. Chen , X. Xu , J. Jiang , Long‐term effect of epigenetic modification in plant–microbe interactions: modification of DNA methylation induced by plant growth‐promoting bacteria mediates promotion process, Vol. 10, Microbiome, Berlin 2022, p. 36; 10.1186/s40168-022-01236-9 PMC8876431 35209943

[12] a) C. Bitterli , G. S. Bañuelos , R. Schulin , J. Geochem. Explor. 2010, 107, 206;

[13] a) P. J. White , M. R. Broadley , New Phytol 2009, 182, 49; 19192191 10.1111/j.1469-8137.2008.02738.x

[14] Q. T. Dinh , Z. Cui , J. Huang , T. A. T. Tran , D. Wang , W. Yang , F. Zhou , M. Wang , D. Yu , D. Liang , Environ. Int. 2018, 112, 294. 29438838 10.1016/j.envint.2017.12.035

[15] C. Lyu , Y. Qin , Z. Zhao , X. Liu , Environ. Pollut. 2021, 273, 116507. 33493758 10.1016/j.envpol.2021.116507

[16] a) B. Zhang , K. Zhou , J. Zhang , Q. Chen , G. Liu , N. Shang , W. Qin , P. Li , F. Lin , Food Chem. 2009, 115, 727;

[17] a) D. Wang , C. Rensing , S. Zheng , J. Hazard. Mater. 2022, 421, 126684; 34339989 10.1016/j.jhazmat.2021.126684

[18] a) R. Yang , Y. He , L. Luo , M. Zhu , S. Zan , F. Guo , B. Wang , B. Yang , Ecotoxicol. Environ. Saf. 2021, 222, 112516; 34273847 10.1016/j.ecoenv.2021.112516

[19] a) S. Clemens , M. G. Aarts , S. Thomine , N. Verbruggen , Trends Plant Sci. 2013, 18, 92; 22981394 10.1016/j.tplants.2012.08.003

[20] Y. V. Nancharaiah , P. Lens , Microbiol. Mol. Biol. Rev. 2015, 79, 61. 25631289 10.1128/MMBR.00037-14 PMC4402961

[21] a) R. K. Sharma , G. Archana , Appl. Soil Ecol. 2016, 107, 66;

[22] a) Y. Gu , S. Banerjee , F. Dini‐Andreote , Y. Xu , Q. Shen , A. Jousset , Z. Wei , ISME J 2022, 16, 2448; 35869387 10.1038/s41396-022-01290-z PMC9478146

[23] P. Mariotte , Z. Mehrabi , T. M. Bezemer , G. B. De Deyn , A. Kulmatiski , B. Drigo , G. C. Veen , M. G. Van der Heijden , P. Kardol , Trends Ecol. Evol. 2018, 33, 129. 29241940 10.1016/j.tree.2017.11.005

[24] J. Zhang , Y.‐X. Liu , X. Guo , Y. Qin , R. Garrido‐Oter , P. Schulze‐Lefert , Y. Bai , Nat. Protoc. 2021, 16, 988. 33442053 10.1038/s41596-020-00444-7

[25] C. Xiong , B. K. Singh , J.‐Z. He , Y.‐L. Han , P.‐P. Li , L.‐H. Wan , G.‐Z. Meng , S.‐Y. Liu , J.‐T. Wang , C.‐F. Wu , Microbiome 2021, 9, 171. 34389047 10.1186/s40168-021-01118-6 PMC8364065

[26] a) Y. Zhou , D. Liu , F. Li , Y. Dong , Z. Jin , Y. Liao , X. Li , S. Peng , M. Delgado‐Baquerizo , X. Li , Nat. Commun. 2024, 15, 6599; 39097606 10.1038/s41467-024-50685-3 PMC11297980

[27] a) A. M. Veach , R. Morris , D. Z. Yip , Z. K. Yang , N. L. Engle , M. A. Cregger , T. J. Tschaplinski , C. W. Schadt , Microbiome 2019, 7, 76; 31103040 10.1186/s40168-019-0668-8 PMC6525979

[28] J. Sasse , E. Martinoia , T. Northen , Trends Plant Sci. 2018, 23, 25. 29050989 10.1016/j.tplants.2017.09.003

[29] H. Feng , R. Fu , J. Luo , X. Hou , K. Gao , L. Su , Y. Xu , Y. Miao , Y. Liu , Z. Xu , New Phytol 2023, 239, 2307. 37357338 10.1111/nph.19086

[30] a) P. J. White , Biochimica et Biophysica Acta (BBA)‐General Subjects 2018, 1862, 2333; 29751098 10.1016/j.bbagen.2018.05.006

[31] J. Kikkert , E. Berkelaar , Arch. Environ. Contam. Toxicol. 2013, 65, 458. 23793939 10.1007/s00244-013-9926-0

[32] Q. Wang , L. Kong , Q. Huang , H. Li , Y. Wan , Front. Plant Sci. 2022, 13, 970480. 36072317 10.3389/fpls.2022.970480 PMC9441932

[33] F. Shimazu , A. Tappel , Science 1964, 143, 369. 14074855 10.1126/science.143.3604.369

[34] B.‐L. Zhang , C.‐C. Guo , F. Ding , Y.‐T. Lu , Z.‐W. Fu , Environ. Exp. Bot. 2019, 163, 69.

[35] a) M. Remelli , V. M. Nurchi , J. I. Lachowicz , S. Medici , M. A. Zoroddu , M. Peana , Coord. Chem. Rev. 2016, 327, 55;

[36] a) Y. Li , R. Cui , P. Zhang , B.‐B. Chen , Z.‐Q. Tian , L. Li , B. Hu , D.‐W. Pang , Z.‐X. Xie , ACS Nano 2013, 7, 2240; 23398777 10.1021/nn305346a

[37] L. Zhang , M. Zhang , S. Huang , L. Li , Q. Gao , Y. Wang , S. Zhang , S. Huang , L. Yuan , Y. Wen , Nat. Commun. 2022, 13, 3361. 35688828 10.1038/s41467-022-31113-w PMC9187771

[38] Z. Cai , T. Yu , W. Tan , Q. Zhou , L. Liu , H. Nian , T. Lian , npj Biofilms Microbiomes 2024, 10, 60. 39043687 10.1038/s41522-024-00532-6 PMC11266425

[39] L. D. Elbourne , S. G. Tetu , K. A. Hassan , I. T. Paulsen , Nucleic Acids Res. 2017, 45, D320. 27899676 10.1093/nar/gkw1068 PMC5210551

[40] E. Bolyen , J. R. Rideout , M. R. Dillon , N. A. Bokulich , C. C. Abnet , G. A. Al‐Ghalith , H. Alexander , E. J. Alm , M. Arumugam , F. Asnicar , Nat. Biotechnol 2019, 37, 852. 31341288 10.1038/s41587-019-0209-9 PMC7015180

[41] D. Ning , M. Yuan , L. Wu , Y. Zhang , X. Guo , X. Zhou , Y. Yang , A. P. Arkin , M. K. Firestone , J. Zhou , Nat. Commun. 2020, 11, 4717. 32948774 10.1038/s41467-020-18560-z PMC7501310

