# Supplementary material for: Dynamic and Stable Core Microbiota Assist Plants in Enriching Selenium and Reducing Cadmium Absorption
Source: Adv Sci (Weinh). 2025 May 19;12(25):e00862. doi: 10.1002/advs.202500862 (PMC12224979; doi:10.1002/advs.202500862)
Supplement: Supplementary file 1 — Supporting Information [file ADVS-12-e00862-s003.docx]

***Supporting Information for***

**Dynamic and stable core microbiota assist plants in enriching selenium and reducing cadmium absorption**

**Author Information**

Zheng Lei ^abc^, Hua Zhang ^b^, Wenju Liu ^c^, Jiandong Sheng ^d^, Huan Zhang ^a^, Yin Wang ^a^, Yanni Tang ^a^, Huaxing Wang ^a^, Cuicui Ding ^a^, Wanqi Qiao ^a^, Yonghui Zhu ^a^, Guoyin Yang ^a^, Yihan Zhang ^a^, Zhuoyi Liu ^a^, Nanyu Zhou ^a^, Chengxiao Hu ^a^, Xiaohu Zhao ^ad*^

*^a^ College of Resources and Environment, Huazhong Agricultural University / Research Center of Trace Elements, Wuhan 430070, China*

*^b^ State Key Laboratory of Environmental Geochemistry, Guiyang 550081, China*

*^c^ State Key Laboratory of North China Crop Improvement and Regulation, Baoding 071001, China*

*^d^ Xinjiang Key Laboratory of Soil and Plant Ecological Processes / College of Resource and Environment, Xinjiang Agricultural University, Urumqi 830052, China*

**Correspondence**: xhzhao@mail.hzau.edu.cn (XH Zhao)

**Supplementary Methods**

**Soil samples pretreatment and measurement of soil properties**

Pre-treat soil of different developmental stages, partially air dry, ground, and sieved through 20 or 100 mesh for the determination of physical and chemical indicators, and partially freeze-drying and then homogenized. The processed samples were then stored at -80 °C. The chemical properties of soils in different developmental stages were measured to monitor the soil nutrient status.

The total Se content in soil and plant was determined as described by Zhang et al(Zhang et al., 2024). Plant samples were digested with an oxidative acid mixture of 9:1 (v/v) HNO_3_:HClO_4_. Soil samples were digested with 20mL HNO_3_ in microwave digestion system (MARS 6 microwave digestion, CEM Corporation, Matthews, NC, USA. Mars X-Press digestion tubes, CEM Corporation, Matthews, NC, USA). For sequential extraction of Se in soil, water-soluble Se (SOL-Se), exchangeable and carbonate-bound Se (EXC-Se), fulvic acid-bound Se (FA-Se), humic acid-bound Se (HA-Se) and residual Se (RES-Se) were sequentially extracted in five steps as described by Zhang et al(Zhang *et al.*, 2024). The Se content of the solution was determined by atomic fluorescence spectrometry (Jitian, Beijing, China). Blank samples were added and returned for analytical quality control. The recovery standard for Se ranges between 90 and 110% was qualified.

The total Cd content in soil and plant was determined as described by Luo et al(Luo et al., 2022). Different tissue samples of plants were digested with HNO_3_-HClO_4_ (5:1, v/v) in microwave digestion system. The Cd fractions (exchangeable Cd, carbonate-bound Cd, Fe-Mn oxides Cd, organic matter-bound Cd, and residual Cd) in rhizosphere soil were analyzed using Tessier's sequential extraction procedure. Cd content (including different fraction) was determined by using graphite furnace atomic absorption spectrometry system (Agilent 240Z AA, CA, USA). The recovery of spiked standard for Cd in plant tissues ranged between 90 and 105%.

**Soil DNA extraction, 16S rDNA amplicon sequencing**

Total microbial community DNA was extracted from the samples using a Fast DNA Spin Kit for Soil (ALFA-SEQ Magnetic Soil DNA Kit). The DNA quality was verified by electrophoresis on a 1% agarose gel. The concentration and purity of the extracted DNA was then confirmed using a NanoDrop 2000 Spectrophotometer (Thermo Fisher Scientific, Inc., USA). The hypervariable region V3-V4 of the bacterial 16S rDNA gene was amplified with primer 338F (5′-ACTCCTACGGGAGGCAGCAG-3′) and 806R (5′-GGACTACHVGGGTWTCTAAT-3′) by PCR thermocycler (BioRad S1000, Bio-Rad Laboratory，CA). PCR conditions were as follows: 5 min at 94°C, then 30 cycles of each 30 s at 94 °C, 30 s at 52 °C and 30 s at 72 °C. After these cycles, there was a 10-min elongation time at 72 °C. Purified amplicons were pooled in equimolar and paired-end sequenced on an Illumina MiSeq PE300 platform (Illumina, San Diego, USA) according to the standard protocols by Meig Smart Technology Co., Ltd (Shenzhen, China). All sequence data have been submitted to the National Center for Biotechnology Information (NCBI) Sequence Read Archive under Bioproject PRJNA1193292.

**Metagenomic sequencing, assembly, and binning**

According to the preliminary results, rhizosphere soil samples were selected to further carry out metagenomics sequencing on an Illumina NovaSeq 6000 platform (Illumina, San Diego, CA) in Majorbio Bio-Pharm Technology Co., Ltd (Shanghai, China). Raw reads were first converted to the fastq. format. The quality of each sample was checked using the FastQC tool; afterward, the adapters were removed using Trimmomatic (v0.39) in KneadData (v0.6.1).

In total, 107.34 Gb raw metagenome sequences (≥ 10.40 Gb per soil sample) were generated from the 9 sequenced samples. Each sample was trimmed, assembled and binned individually according to the metaWRAP pipeline (v1.2.1)(Uritskiy et al., 2018). The raw reads were first trimmed with the Read_qc module to remove low-quality reads and then assembled into contigs using MEGAHIT (v1.1.3) with various *k*-mer sizes (ranging from 21 to 141)(Li et al., 2015a). The non-redundant genes were annotated using KEGG and COG databases by eggNOG-mapper (v2.1.6) and using CAZy database by DIAMOND. Multiple comparisons of amino acid metabolism processes at different developmental stages were conducted based on the KEGG database. Only contigs ≥ 1000 bp were kept for subsequent analysis. The assembled contigs from each sample were individually binned using Metabat2 (v2.12.1) and MaxBin2 (v2.2.7)(Kang et al., 2015; Wu et al., 2016). The original bins were consolidated and improved with Bin_refinement and Reassemble_bins modules in metaWRAP. The quality of the bins was evaluated with CheckM (v1.0.12)(Parks et al., 2015). Bins with estimated genome completeness >50% and contamination <10% were kept as metagenome-assembled genomes (MAGs), and we finally obtained 22 bacterial MAGs for subsequent analysis.

**Growth-promoting properties of Se/Cd related rhizoplane bacteria**

To understand the growth-promoting properties of Se/Cd related bacterial cluster members, the ability of nitrogen fixation, phosphorus solubilization (organic phosphorus and inorganic phosphorus), siderophore, potassium solubilization, ACC synthase production, IAA production of these strains were determined (Fig. 5b). Briefly, Azotobacter, Pikovskaya, and CAS agar plates were added with two microliters of bacterial suspension and cultured at 28 °C for 5 days. Clear zones were observed on Azotobacter, Pikovskaya and organic phosphorus agar medium plates, and yellow-orange halo were observed on CAS plates, indicating that the strain had the ability of nitrogen fixation, phosphorus solubilization, and siderophore production, respectively. For IAA production, strains were cultivated in a TSB medium containing 5 mmol/L tryptophan. After culturing at 28 °C for 48 h, supernatant was obtained by centrifugation at 10,000 r/min for 5 min. One milliliter supernatant with the same amount of Salkawski chromogenic agent were placed in darkness for 30 min for the IAA chromogenic reaction. The color of the solution changes to pink as a positive reaction that the strains produce IAA(Zhou et al., 2024). For ACC deaminase activity, *AcdSf3*/*AcdSr4* primers were used to identify ACC deaminase producing bacteria(Li et al., 2015b).

To evaluate the selenium reduction and cadmium adsorption abilities of the isolated selenium cadmium related strains, we added an additional 0.1 mM Na_2_SeO_3_ and/or 0.1 mM CdCl_2_ to the TSB culture medium, inoculated the selenium cadmium related strains, centrifuged the culture medium after 5 days, and measured the selenium/cadmium concentration in the supernatant. Compare with the control group(no-inoculation) to calculate the Se reduction rate and Cd adsorption rate of the strain.

Additionally, pot experiments were conducted to explore the effects of synthetic microbial communities and key glutathione - related amino acids on plant growth in natural soils with Se - and Cd - coexistence. Three natural soils with varying Se and Cd concentrations were used, with 500g per pot. After surface - sterilizing the seeds and disinfecting them, they were placed in a seedling tray to germinate for 7 days. Seedlings with uniform growth were then transferred to the pots. The 14 - days pot experiments were conducted in the greenhouse with a 12 h: 12 h, light: dark (26°C-28°C), and the pots were randomly replaced at regular intervals to ensure the same growth conditions. There were two treatments: Control (ddH₂O addition) and A+S (applying amino acids related to the glutathione metabolic pathway and inoculating synthetic microbial communities). After the pot experiment, collect plant shoots and root samples to measure fresh and dry weights.

In addition, we conducted a plant agar culture experiment with the same treatments as the pot experiment. After sterilizing and cooling the one - half strength MS agar medium, surface - sterilized rapeseed seeds were inoculated onto the agar plates at the same horizontal position. Agar culture experiments were conducted in a plant incubator under a 12 h light/dark cycle at 26°C - 28°C. After 7 - days, plant samples were collected and root lengths were measured.

**Determination of glutathione metabolic pathway gene expression in strains**

We selected isolated strain *Phenylobacterium zucineum* (ASV26) from the synthetic community as the test strain because it had the highest relative abundance among strains in the positive group that showed a strong positive correlation with available selenium and a strong negative correlation with available cadmium. Four treatments were set: 1) LB medium (Control); 2) LB medium with 0.1 mM Na₂SeO₃ and 0.1 mM CdCl₂ (SeCd); 3) SeCd treatment with added glutathione - related amino acids (SeCd + Amino acid); and 4) SeCd + Amino acid treatment with added glutathione synthesis inhibitor (SeCd + Amino acid + BSO). We selected target genes including *gisA*, *gsiB*, *ArgT* (amino acid transport), *gltL*, *gltK* (glutathione transport), *gshA*, *gshB* (glutathione synthesis), and *gor*, *ggt* (glutathione utilization and degradation).

The strain was cultured in LB media shaking until logarithmic growth. Total RNA was extracted using TRIzol (Invitrogen), with integrity and concentration assessed via 1% agarose gel electrophoresis and a NanoDrop spectrophotometer. cDNA was synthesized from RNA using PrimeScript™ RT Master Mix in a 20-μL reaction containing 4 μL 5X PrimeScript RT Master Mix, RNA, and RNase-free water, incubated at 37°C for 15 minutes and 85°C for 5 seconds, and stored at -20°C. qPCR was performed in a 20-μL system with 10 μL 2X Hieff® qPCR SYBR Green Master Mix (No Rox, Yeasen), 0.4 μL each of forward and reverse primers (10 μM), diluted cDNA, and RNase free water. Reactions were run on a CFX Opus 384 Real-Time PCR System (Bio-Rad) with 95°C pre-denaturation for 3 minutes, 45 cycles of 95°C for 10 seconds and 60°C for 30 seconds, and a melting curve analysis. Data were analyzed using the 2^-ΔΔCt method, normalizing target gene expression to the reference gene (16S gene and *recA* gene) Ct values, via CFX Manager software (Bio-Rad).

Primers sequence (5'-3') for these genes are shown below: *16S* (515-F:GTGCCAGCMGCCGCGGTAA; 806R:GGACTACHVGGGTWTCTAAT), *recA* (recA-F:ATGACTCAGTCGGCGTTGAGG; recA-R:TCAGAGCGAGCCGTCGGC), *gshA* (gshA-F:ATGGCGCTTGAGGTCGTCTCCAC; gshA-R: CTAGGCGAGGCGCTCGGCGT), *gshB* (gshB-F:ATGTCGCTGAAGGTCGCCGTCCAGATG; gshB-R:CTAGGCCCCGGCGCGAAGC), *gor* (gor-F:TTGGCCGGATACGACTACG; gor-R:TCAGGCCACCTGACCCAG), *ggt* (ggt-F:CTGGATCGAGCTGGACGTG; ggt-R: TCAGGCGATGCCGAGCGC) , *TcyC* (TcyC -F:ATGGCGCTGGGGAGCGCA; TcyC -R:CTACCGGAGGCTGGCGAG), *gltL* (gltL-F: ATGGCCGATGACGCCGCCTACGTC; gltL-R:TCAGCTCGCGGCCGCGCC) , *gltK* (gltK-F:ATGACCCCCGATCCGCGCGATCC; gltK-R:CTACGCTCCCGCGCGCACC) , *ArgT* (ArgT-F: CCGAATCGCTGAAGGGCAAG; ArgT-R:CCTGGTTCTGGTCCTGGTACG), *GsiA* (GsiA-F: ATGAAGCTGTACTACGCTCCAGGC; GsiA-R:TCAGGCCGCCGCCTGCTC), *GsiB* (GsiB-F: ATGATCACCCTGCATGGACGCG; GsiB-R:CTACGCAGCCTGGGCCGC)

**Reference**

**Kang, D.D., Froula, J., Egan, R., and Wang, Z.** (2015). MetaBAT, an efficient tool for accurately reconstructing single genomes from complex microbial communities. PeerJ **3**:e1165.

**Li, D., Liu, C.-M., Luo, R., Sadakane, K., and Lam, T.-W.** (2015a). MEGAHIT: an ultra-fast single-node solution for large and complex metagenomics assembly via succinct de Bruijn graph. Bioinformatics **31**:1674-1676.

**Li, Z., Chang, S., Ye, S., Chen, M., Lin, L., Li, Y., Li, S., and An, Q.** (2015b). Differentiation of 1-aminocyclopropane-1-carboxylate (ACC) deaminase from its homologs is the key for identifying bacteria containing ACC deaminase. FEMS Microbiology Ecology **91**:fiv112.

**Luo, J., Gu, S., Guo, X., Liu, Y., Tao, Q., Zhao, H.-P., Liang, Y., Banerjee, S., and Li, T.** (2022). Core microbiota in the rhizosphere of heavy metal accumulators and its contribution to plant performance. Environmental Science & Technology **56**:12975-12987.

**Parks, D.H., Imelfort, M., Skennerton, C.T., Hugenholtz, P., and Tyson, G.W.** (2015). CheckM: assessing the quality of microbial genomes recovered from isolates, single cells, and metagenomes. Genome research **25**:1043-1055.

**Uritskiy, G.V., DiRuggiero, J., and Taylor, J.** (2018). MetaWRAP—a flexible pipeline for genome-resolved metagenomic data analysis. Microbiome **6**:1-13.

**Wu, Y.-W., Simmons, B.A., and Singer, S.W.** (2016). MaxBin 2.0: an automated binning algorithm to recover genomes from multiple metagenomic datasets. Bioinformatics **32**:605-607.

**Zhang, H., Yang, D., Hu, C., Du, X., Liang, L., Wang, X., Shi, G., Han, C., Tang, Y., and Lei, Z.** (2024). Bacteria from the rhizosphere of a selenium hyperaccumulator plant can improve the selenium uptake of a non-hyperaccumulator plant. Biology and Fertility of Soils **60**:987-1008.

**Zhou, Y., Liu, D., Li, F., Dong, Y., Jin, Z., Liao, Y., Li, X., Peng, S., Delgado-Baquerizo, M., and Li, X.** (2024). Superiority of native soil core microbiomes in supporting plant growth. Nature communications **15**:6599.

**Supplementary Results**

### Community assembly processes across the entire plant developmental cycle in soils with different levels of Se and Cd

Exploring the process of community assembly across plant development is crucial for gaining a deeper understanding of the microbial community responses and feedback driven by plant development in soils with different levels of Se and Cd. To this end, phylogenetic bin-based null model analysis (iCAMP) was used to quantify the potential contributions of deterministic processes (homogeneous and heterogeneous selection) and stochastic processes (diffusion limitation, homogeneous diffusion, and drift) in the bacterial community assembly (Fig. S5). The results indicated that drift was the most important process shaping the dynamic rhizosphere microbial communities across the different developmental stages of the plants. The average proportion of the deterministic processes of these rhizosphere microbial communities showed an increasing trend with increasing Se and Cd content (Lsoil: 18.0%, Msoil: 19.6% and Hsoil: 24.8%). This trend was more pronounced during the flowering period (Lsoil: 15.9%, Msoil: 21.2%, and Hsoil: 26.9%).

**Supplementary Figures**


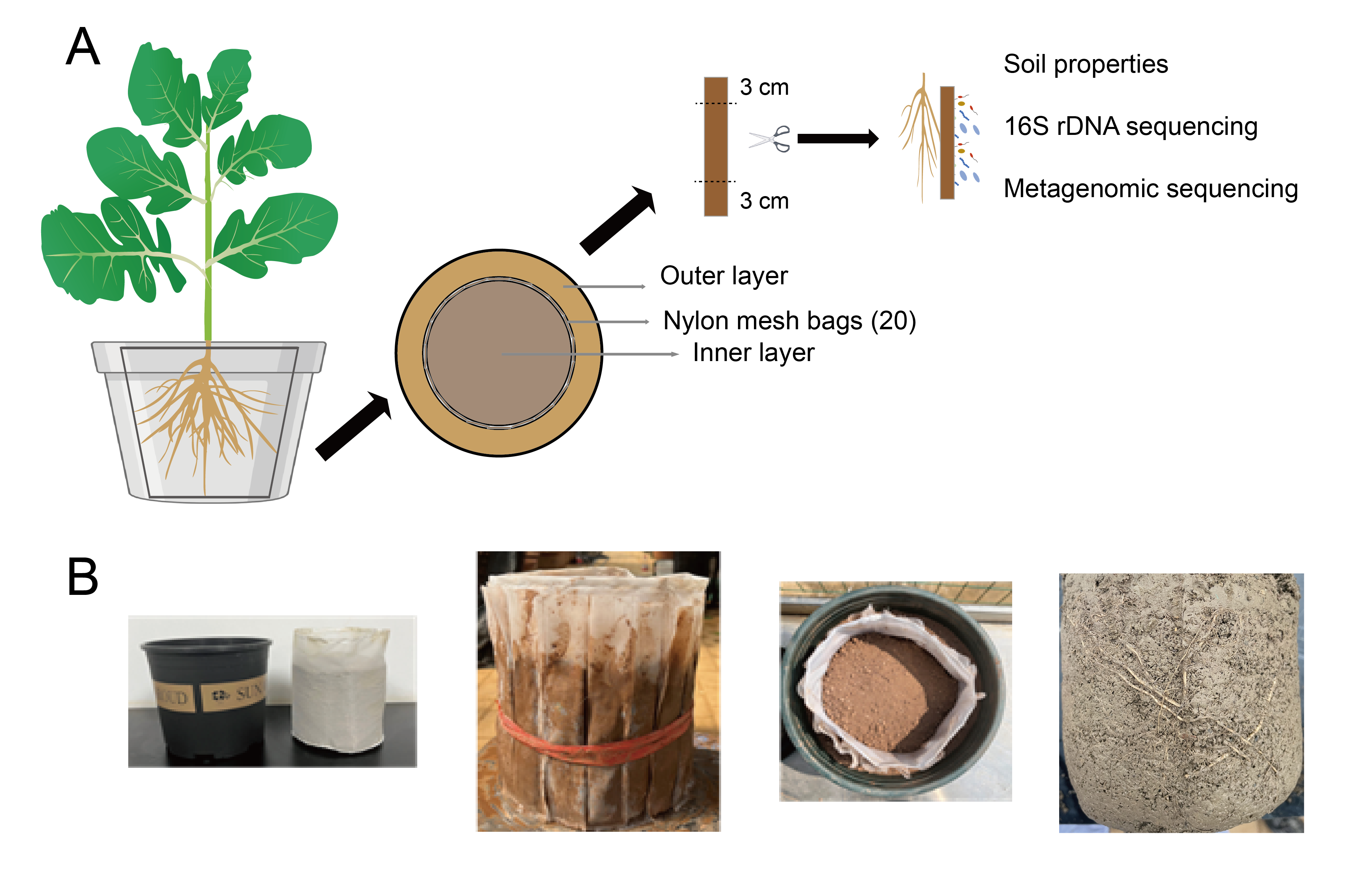


**Supplementary Fig. 1 Schematic figure of the rhizobox sampling system and the experimental design.** A) The “rhizobox” consisted of a three-layer cylinder, The inner layer (root compartment) is made of a 50-μm nylon mesh net, which prevents roots from entering into the middle layer. The middle sampling layer consisted of 20 individual nylon mesh bags (150 μm nylon mesh. During each sample collection, two nylon bags were randomly selected, and the top 3 cm and bottom 3 cm were trimmed off, with soil from the middle section being collected. The soil in the nylon mesh bags of the middle layer was thus in close contact with plant roots and root exudates and was used as a proxy of rhizosphere bacterial community. B) Experimental setup photo. The central root compartment was densely colonized by plant roots.


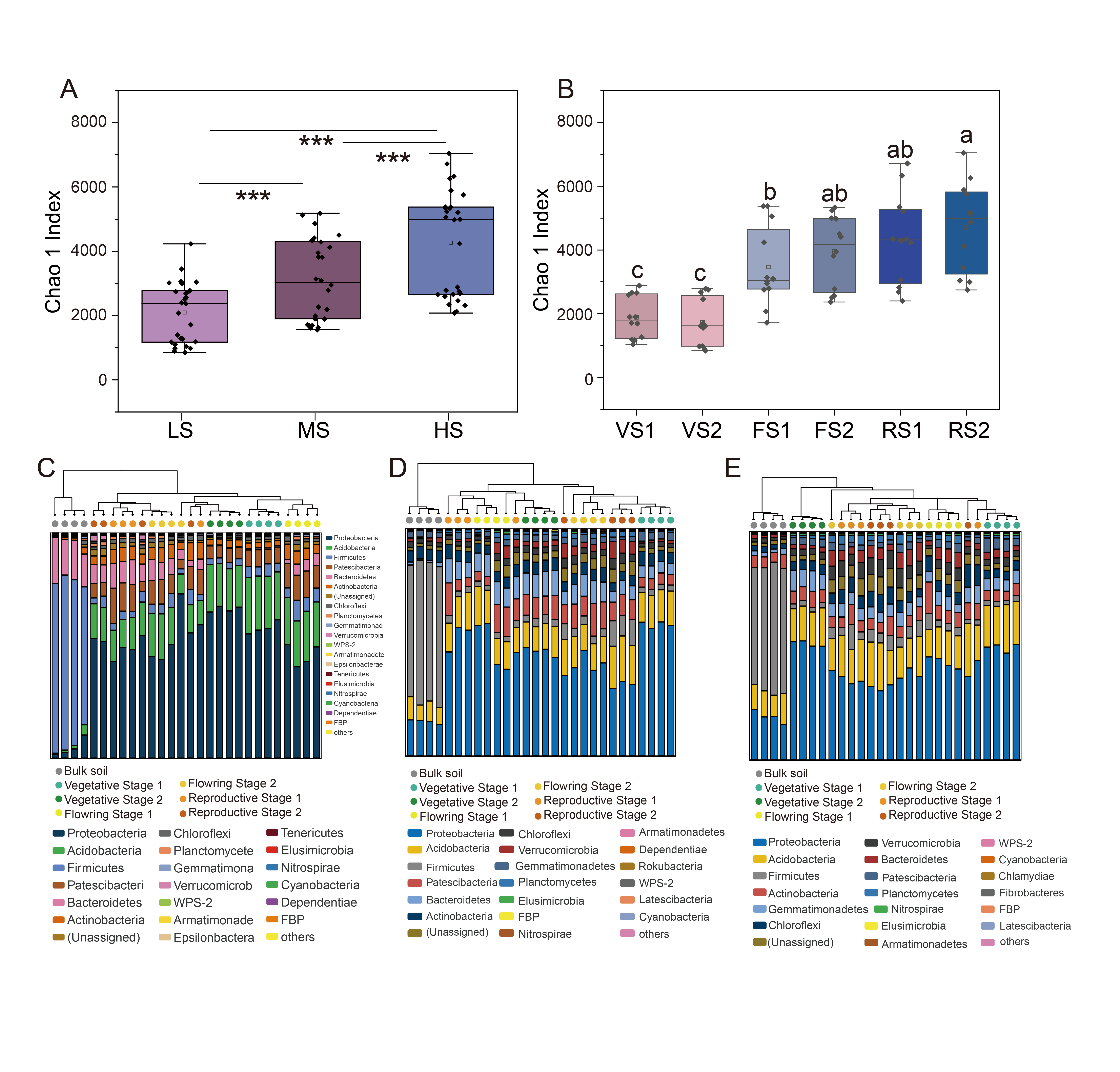


**Supplementary Fig. 2 α-diversity and community composition of rhizosphere microbes across three types of soils and different developmental stages.** A) rhizosphere microbial α-diversity in three types of soils, including all samples from the entire growth stage. The asterisks represent the level of significance (**P* < 0.05, ***P* < 0.01, ****P* < 0.001) among different samples based on one-way ANOVA test with Dunnett’s post hoc analysis. B) α-diversity changes of rhizosphere microbes across various developmental stages. Each time point includes all samples from the three types of soils at that time point.Different letters indicate significant difference of plant growth phenotypes in different treatments at *P* < 0.05 by one-way ANOVA test. **(c-e)** Cluster analysis of community composition for plants in three soils at all time points across different developmental stages. From left to right, they represent soils with low, medium, and high Se-Cd content, respectively.


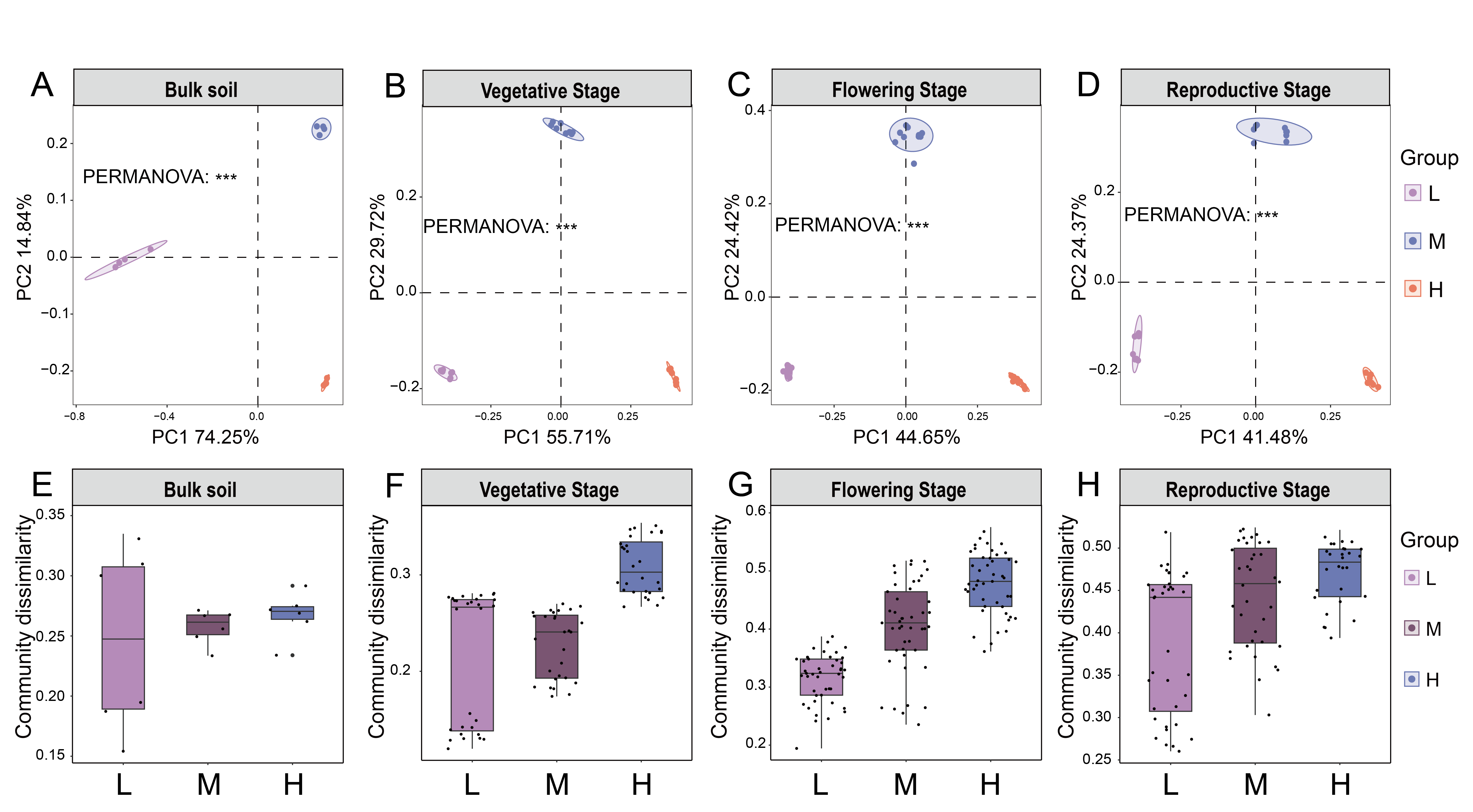


**Supplementary Fig. 3 Diversity analysis and dissimilarity analysis of rhizosphere microbial communities.** A-D) PCA analysis of rhizosphere microbial communities at different developmental stages for plants grown in three types of soils with varying Se-Cd content, where L, M, and H represent low, medium, and high Se-Cd content soils, respectively. The asterisks represent the level of significance (**P* < 0.05, ***P* < 0.01, ****P* < 0.001) among different samples based on one-way ANOVA test with Dunnett’s post hoc analysis. E-H) Community dissimilarity of rhizosphere microbial communities at different developmental stages for plants grown in three types of soils with varying Se-Cd content, where L, M, and H represent low, medium, and high Se-Cd content soils, respectively.


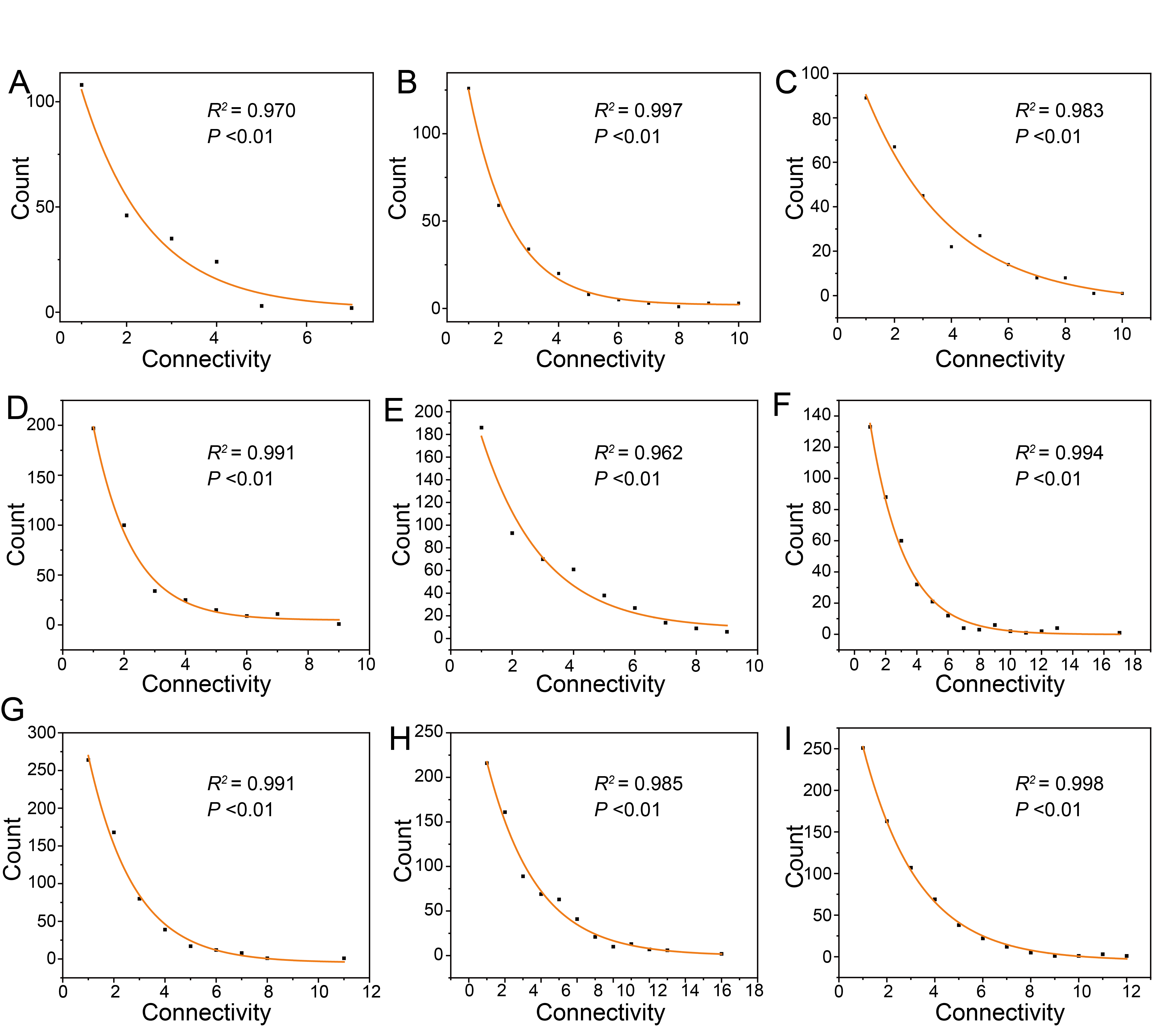


**Supplementary Fig. 4 Degree distributions of nodes in the nine microbial ecological networks.** A-C) The rhizosphere microbial network of plants at different developmental stages in Lsoil. D-F) The rhizosphere microbial network of plants at different developmental stages in Msoil. G-I) The rhizosphere microbial network of plants at different developmental stages in Hsoil. *R*^2^ represents the goodness of fit of a power-law model.


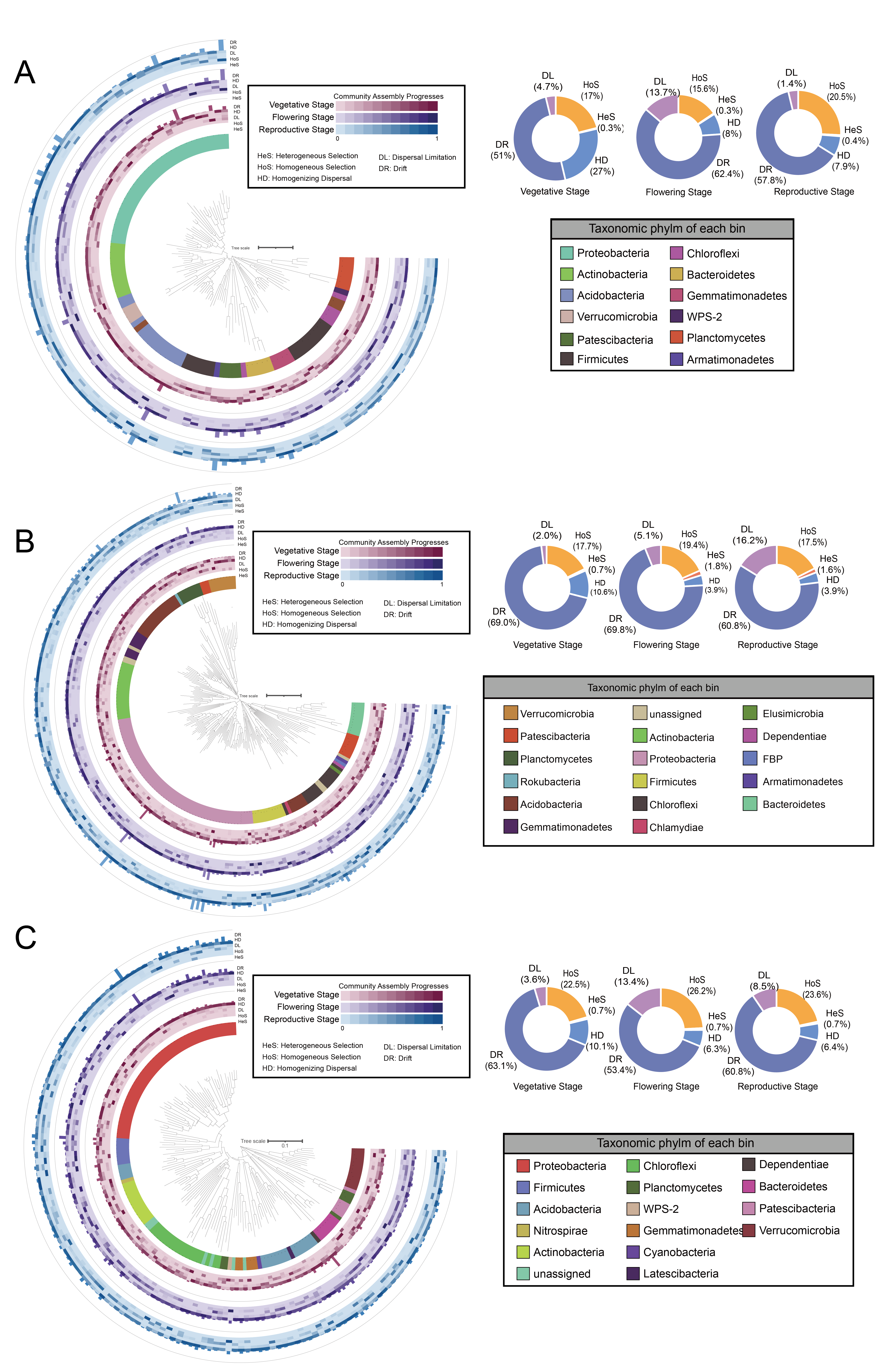


**Supplementary Fig. 5 Variations of ecological processes across different phylogenetic groups.** A-C) Phylogenetic tree was displayed at the center. Homogeneous selection (HoS); heterogeneous selection (HeS); homogenizing dispersal (HD); dispersal limitation (DL); drift (DR). The first circle represents the systematic classification of all Bins at the phylum level, and the second to fourth circles represent the proportion of community assembly processes of each Bin during the vegetative stage, flowering stage, and reproductive stage, respectively. The three figures from top to bottom describe the community assembly in soils with low, medium, and high Se-Cd enrichment.


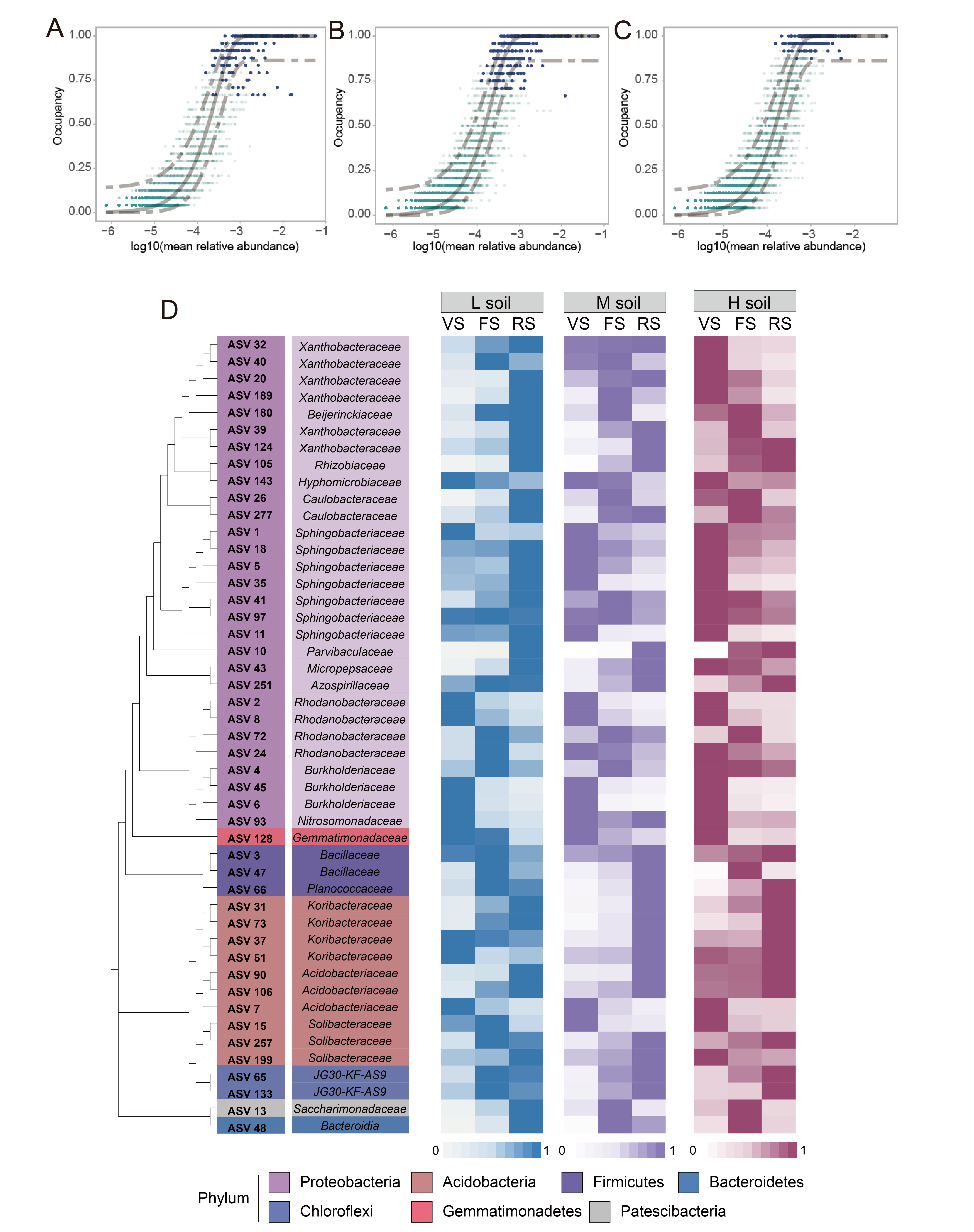


**Supplementary Fig. 6 Identification and systematic classification analysis of core microbes that persistently and stably exist in three dynamic rhizosphere soils.** A-C) Occupancy–abundance curves for rhizosphere communities of three Se-Cd rich soil. X-axis displays the log-transformed mean relative abundance of each ASV, y axis displays percentage of samples in which each ASV was detected. D) Phylogenetic tree and phylum-level classification of core microbial members that are stably present across all developmental stages in the three soils, with the heatmap on the right showing the abundance changes of these ASVs at different developmental stages in the three soils.


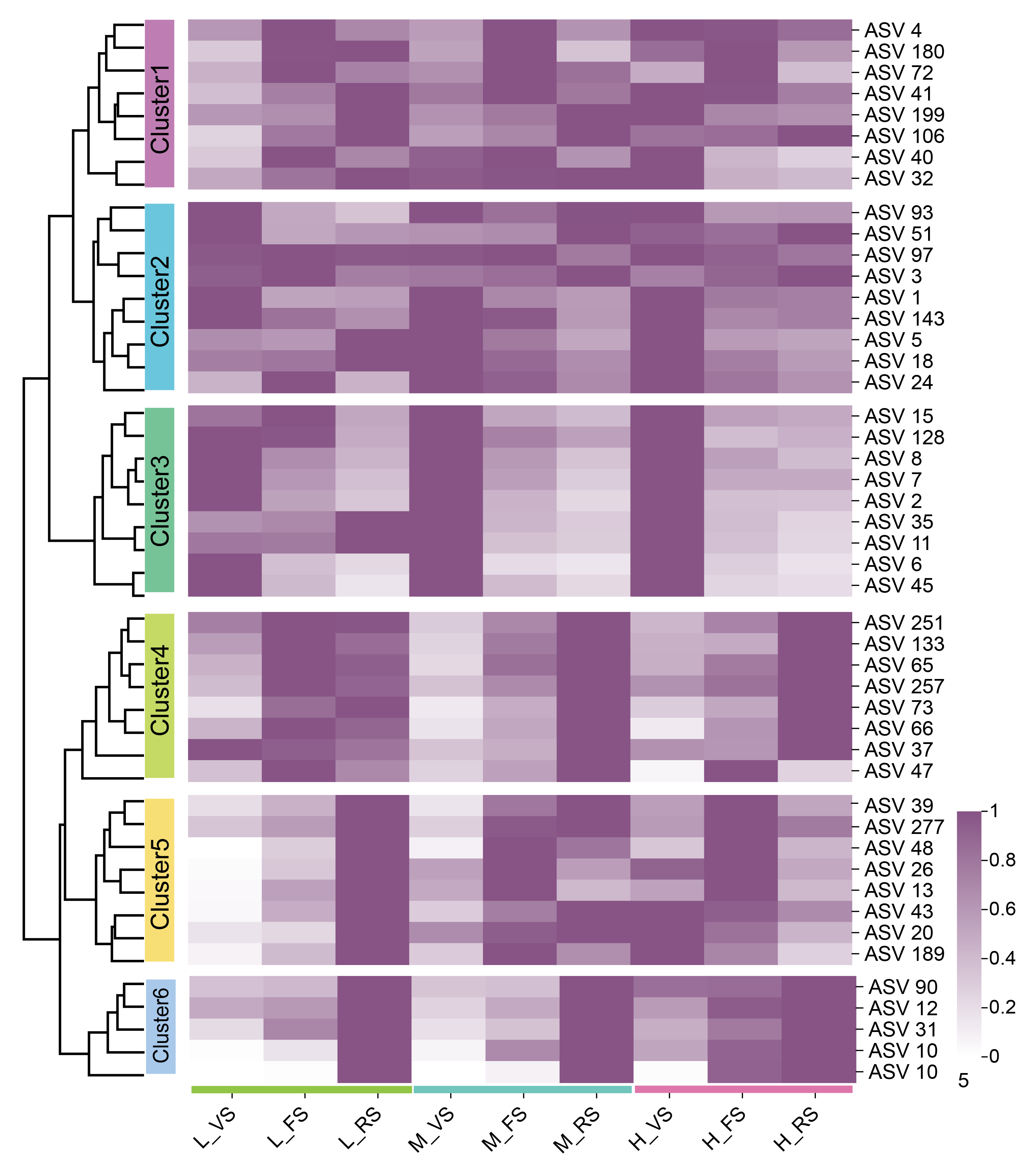


**Supplementary Fig. 7 Cluster analysis based on relative abundance revealed the core microbial members**. These ASVs were categorized into five distinct clusters**. ‘**L’, ‘M’, and ‘H’ represent low, medium, and high Se-Cd content soils, respectively.


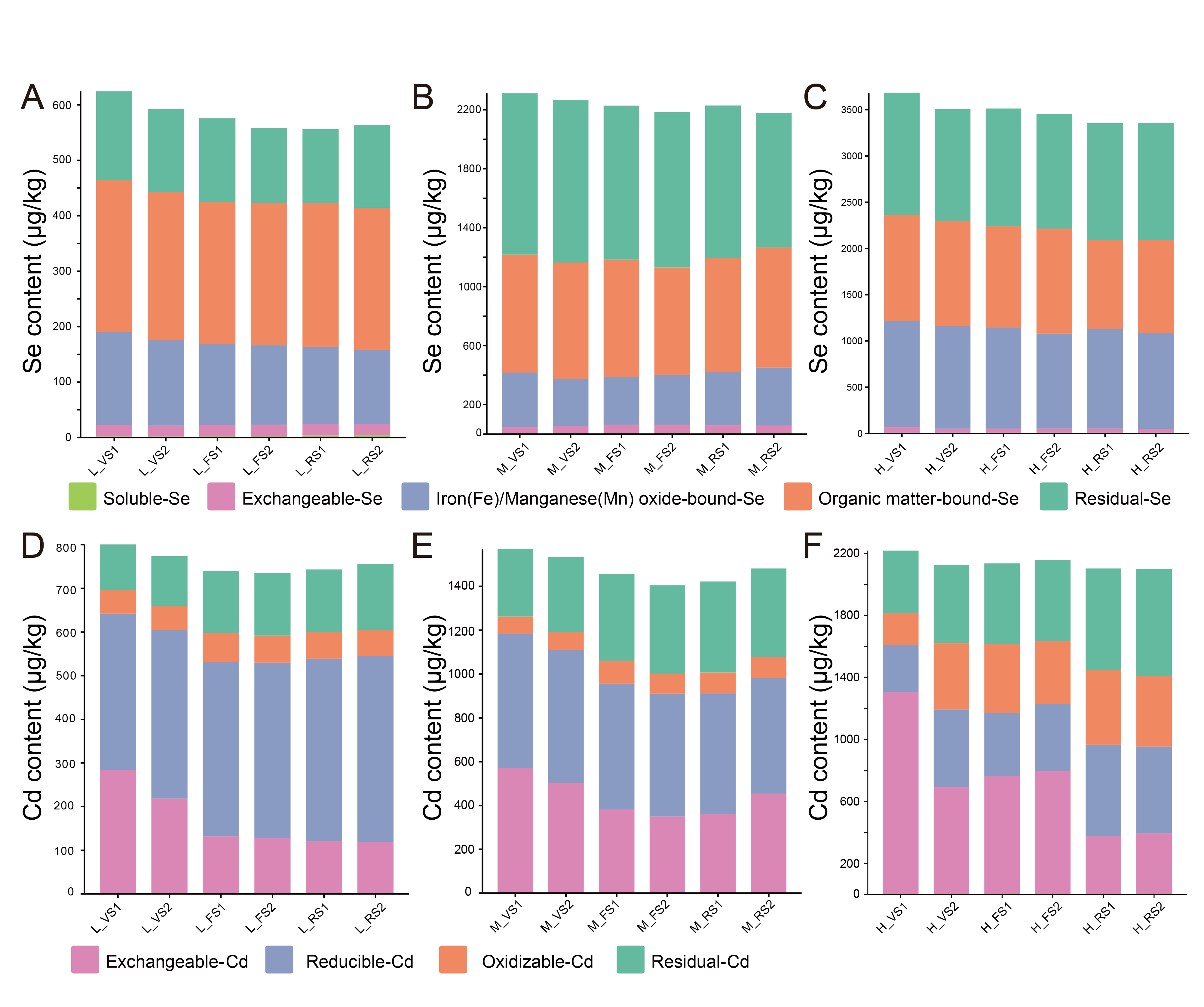


**Supplementary Fig. 8 The fraction of Se and Cd speciation in rhizosphere soil at different developmental stages of plants.** A-C) Panels a, b, and c represent the fraction of Se in rhizosphere soil for low, medium, and high Se-Cd rich soils, respectively. D-F) Panels d, e, and f represent the fraction of Cd in rhizosphere soil for low, medium, and high Se-Cd rich soils, respectively. **‘**L’, ‘M’, and ‘H’ represent low, medium, and high Se-Cd content soils, respectively.


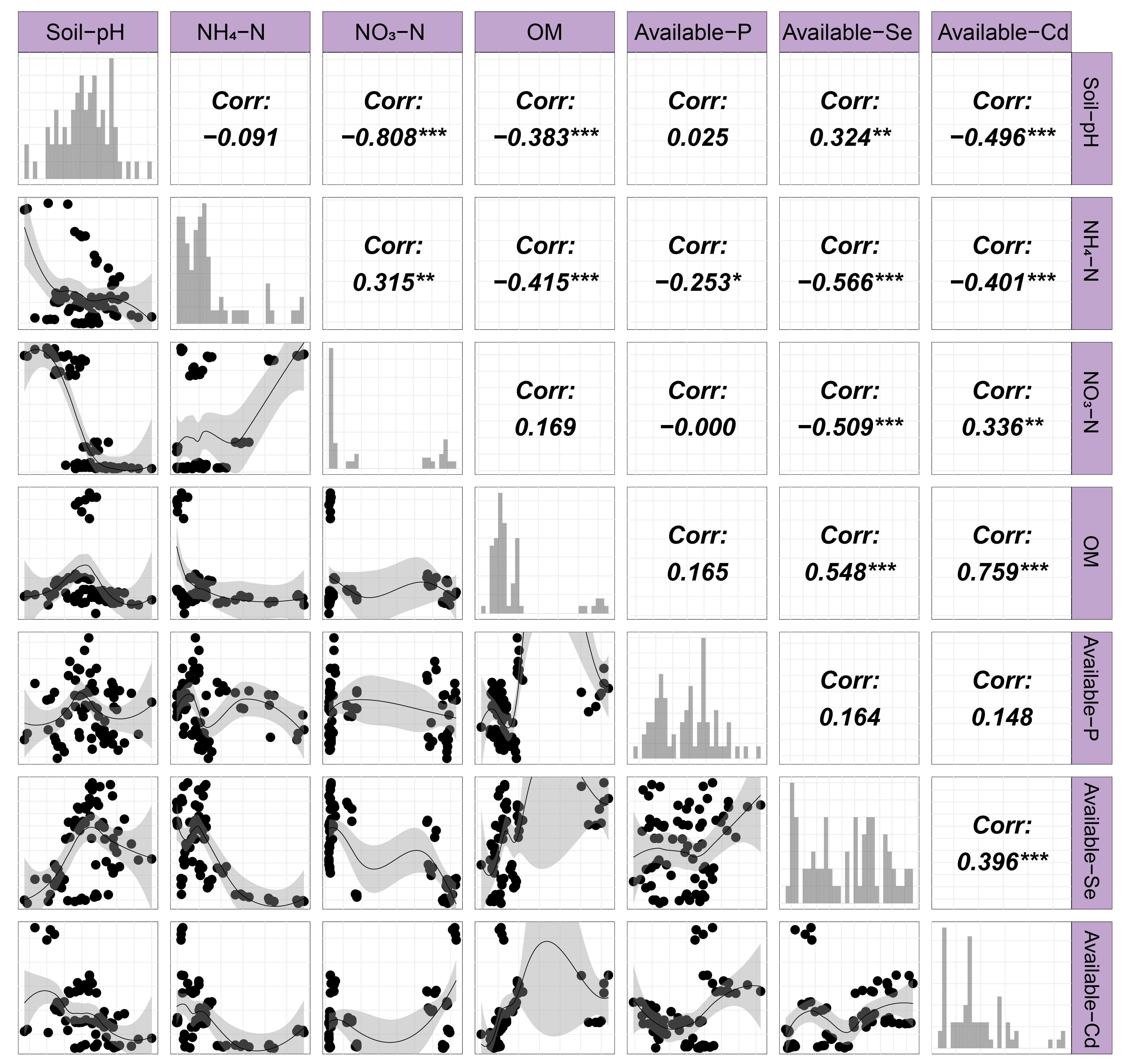


**Supplementary Fig. 9 Correlation matrix diagram of rhizosphere soil environmental factors across the plant growth cycle.** The analysis was conducted using the Spearman method. The scatter plots in the lower left represent measured values of different environmental factors, the histograms in the middle show the density of these factors, and the correlation and significance between environmental factors are indicated in the boxes in the upper right. Each index was measured with four biological replicates.


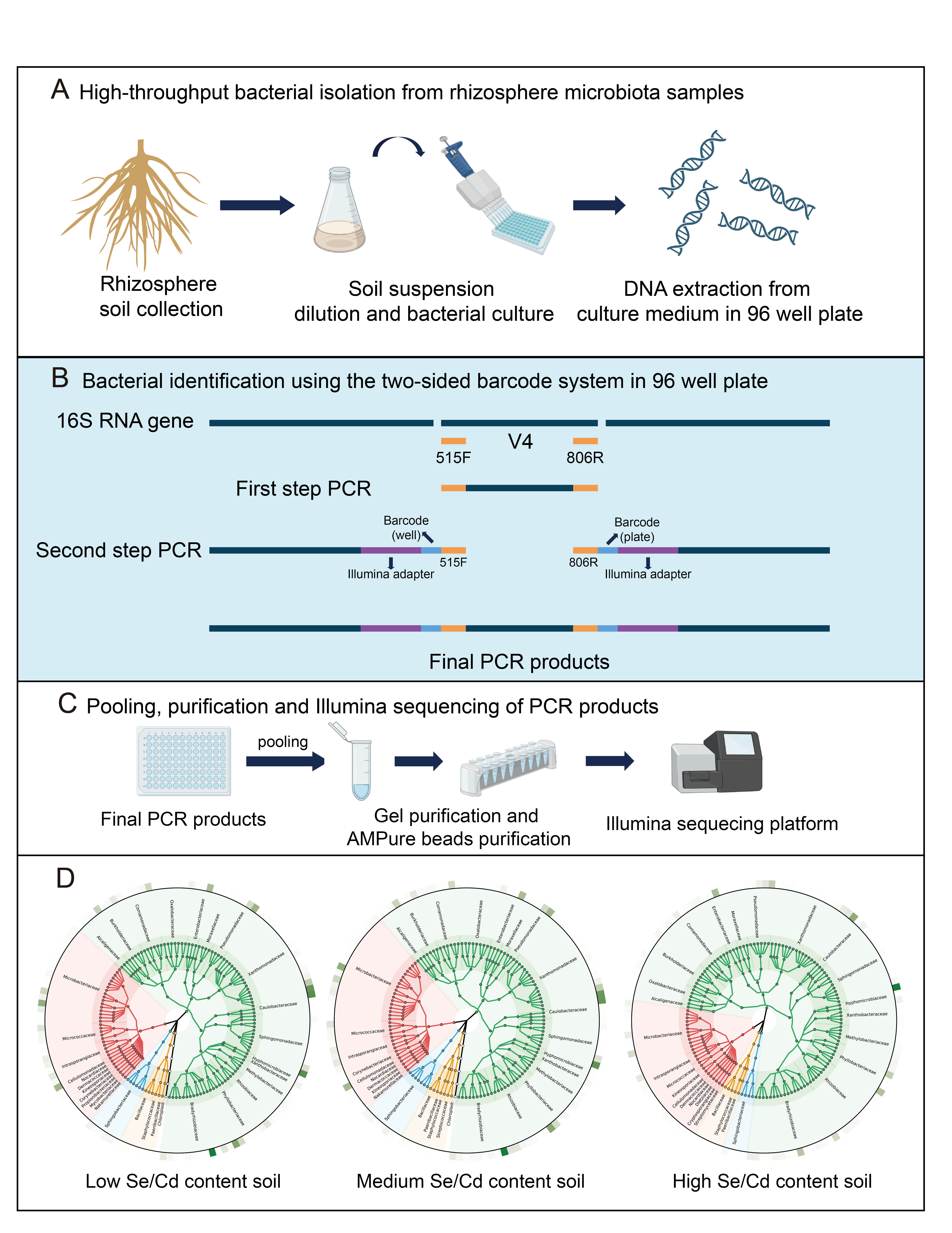


**Supplementary Fig. 10 Overview of the high-throughput bacterial cultivation and identification system.** A) High-throughput bacterial isolation from root microbiota samples and DNA extraction. B) Bacterial identification using the two-sided barcode system in 96 well plate. C) Pooling, purification and illumina sequencing of PCR products. D) Phylogenetic tree of bacteria isolated from the rhizosphere of rapeseed grown in three types of naturally Se-Cd enriched soils.


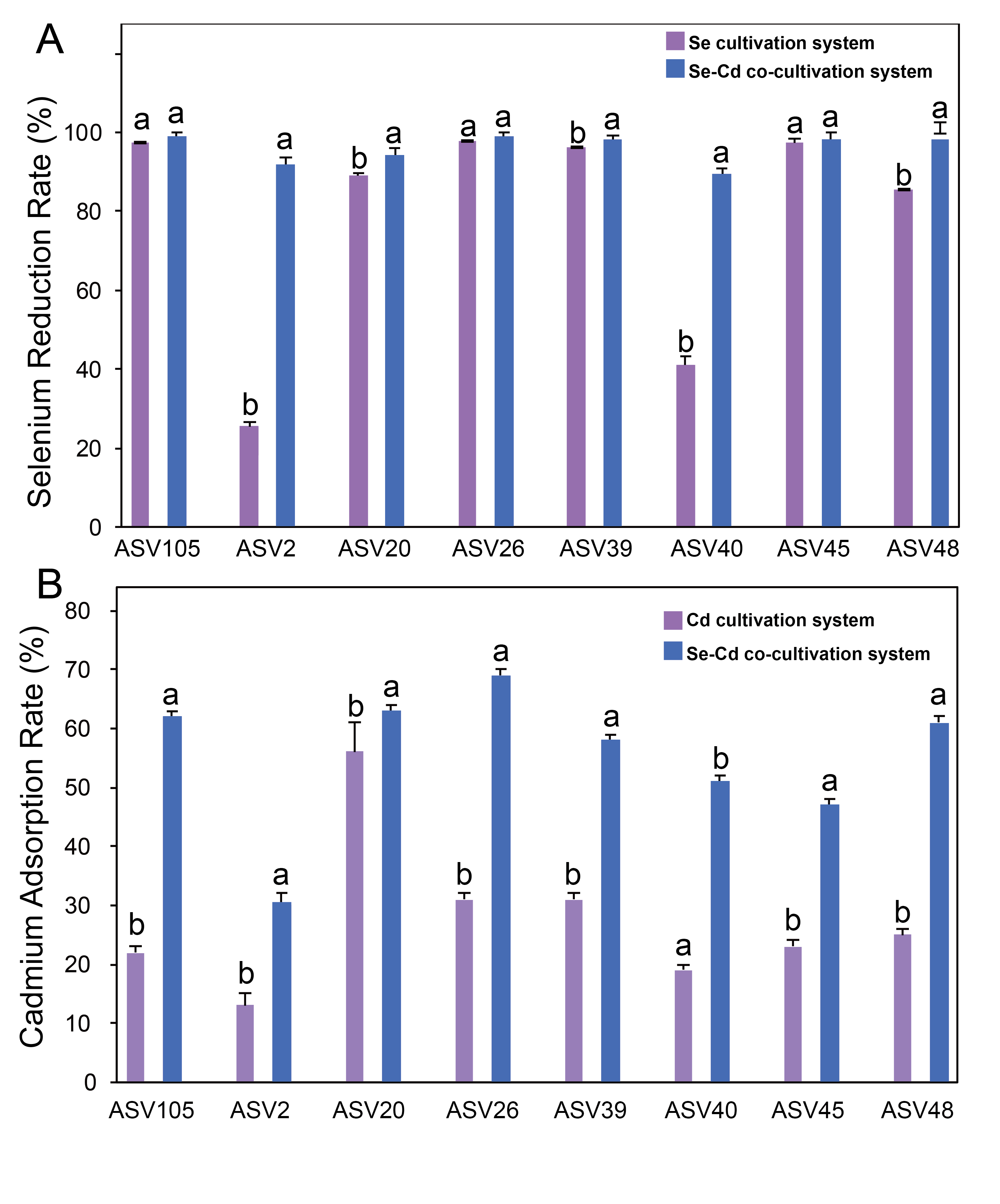


**Supplementary Fig. 11 Determination of Se reduction and Cd adsorption capacities of Se-Cd-related key strains.** A) Measurement of selenium reduction capacity. B) Measurement of cadmium adsorption capacity. Different letters indicate significant differences among different treatments at P < 0.05 by one-way ANOVA test.


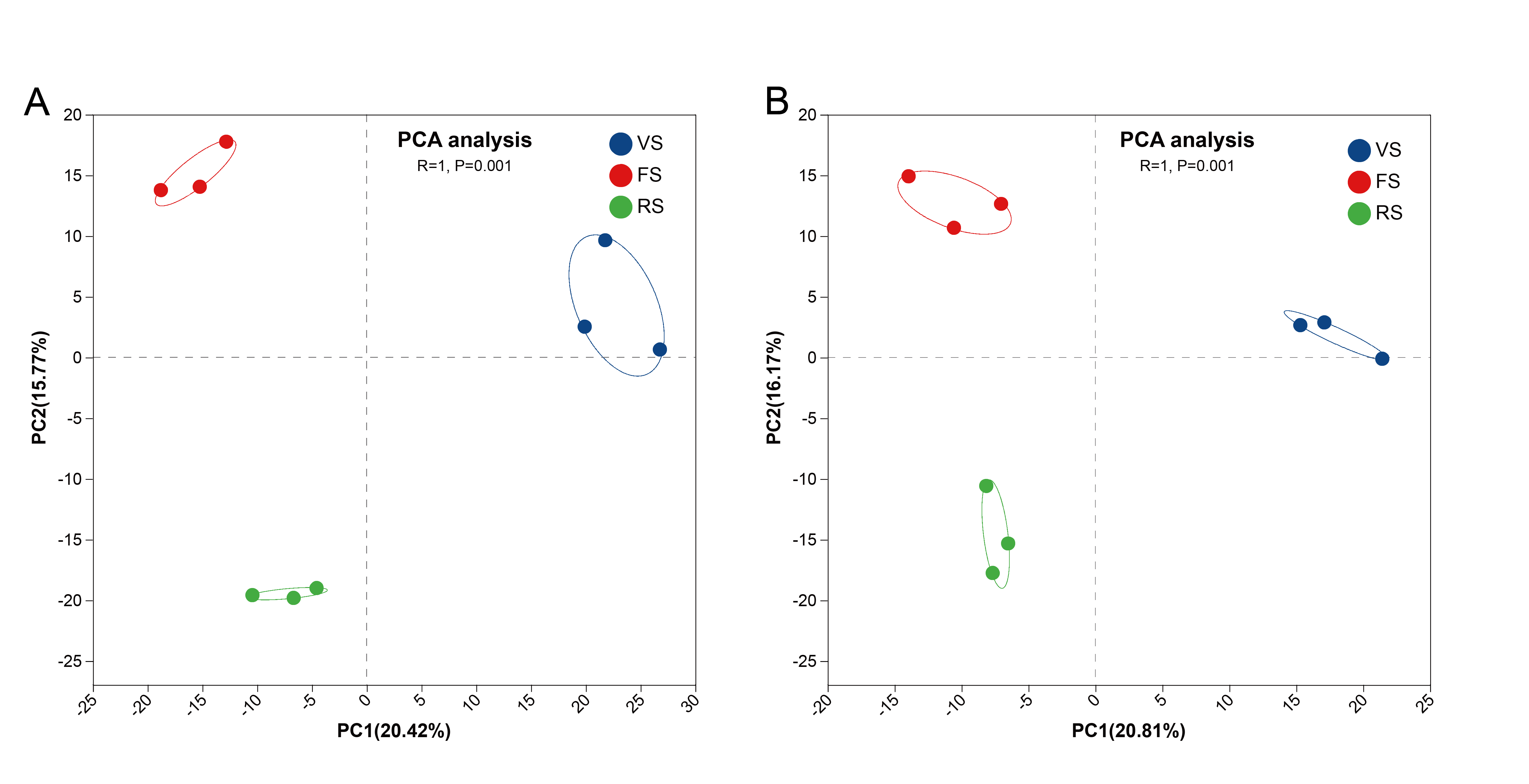


**Supplementary Fig. 12 Metagenomic PCA analysis based on the NR database.** A) Untargeted metagenomic analysis, which includes all non-redundant gene sets. B) Targeted metagenomic analysis, which includes gene sets known to be associated with soil Se cycling and Cd uptake, binding, and excretion.


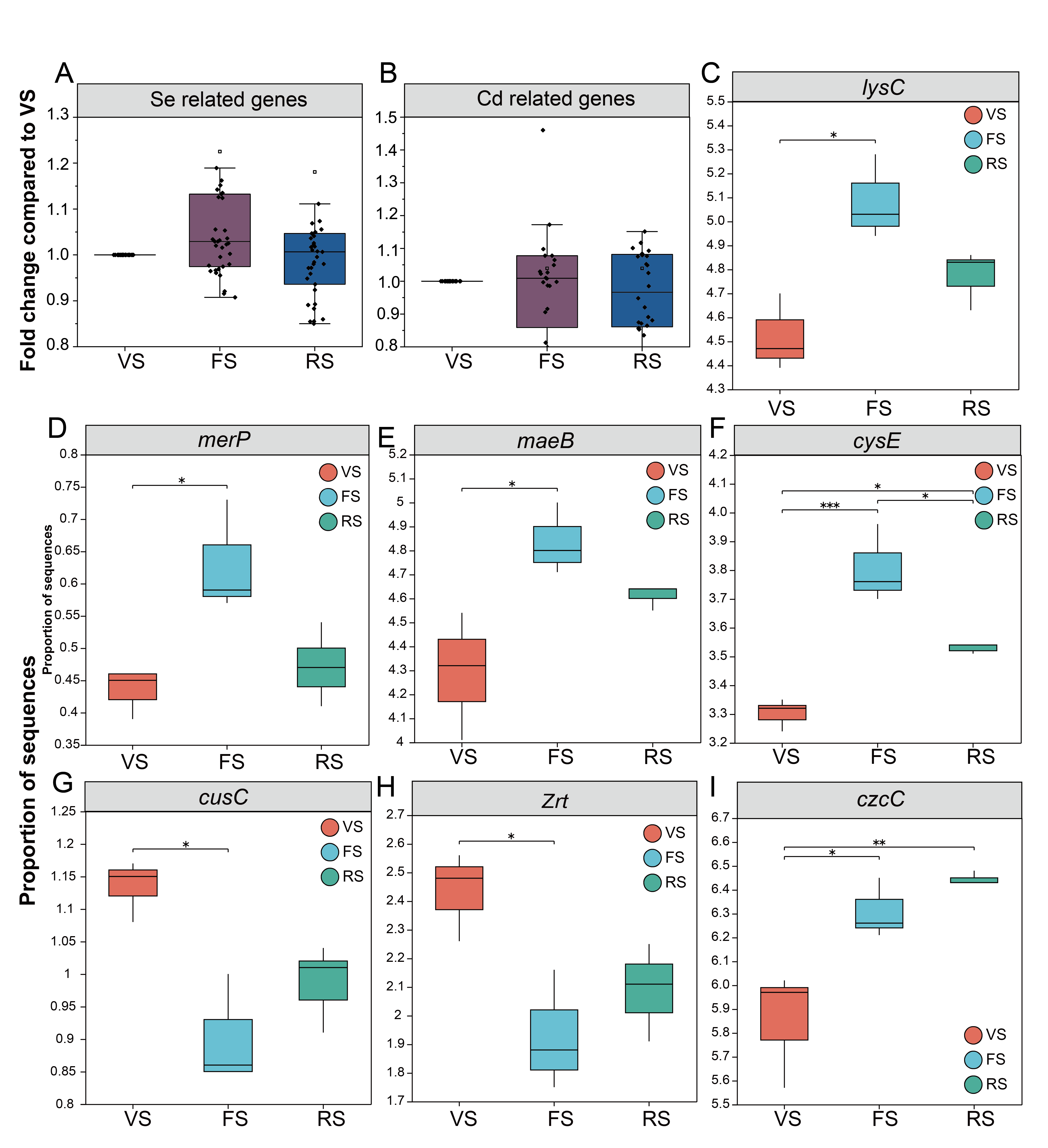


**Supplementary Fig. 13 Metagenomic targeted analysis of Se and Cd-related genes.** Fold change of Se (A) and Cd (B) related genes in the rhizosphere soil of plants compared to the VS stage. C-I) Gene abundance of Se and Cd-related genes in the rhizosphere soil at different stages of plant development. The asterisks represent the level of significance (**P* < 0.05, ***P* < 0.01, ****P* < 0.001) among different samples based on one-way ANOVA test with Dunnett’s post hoc analysis.


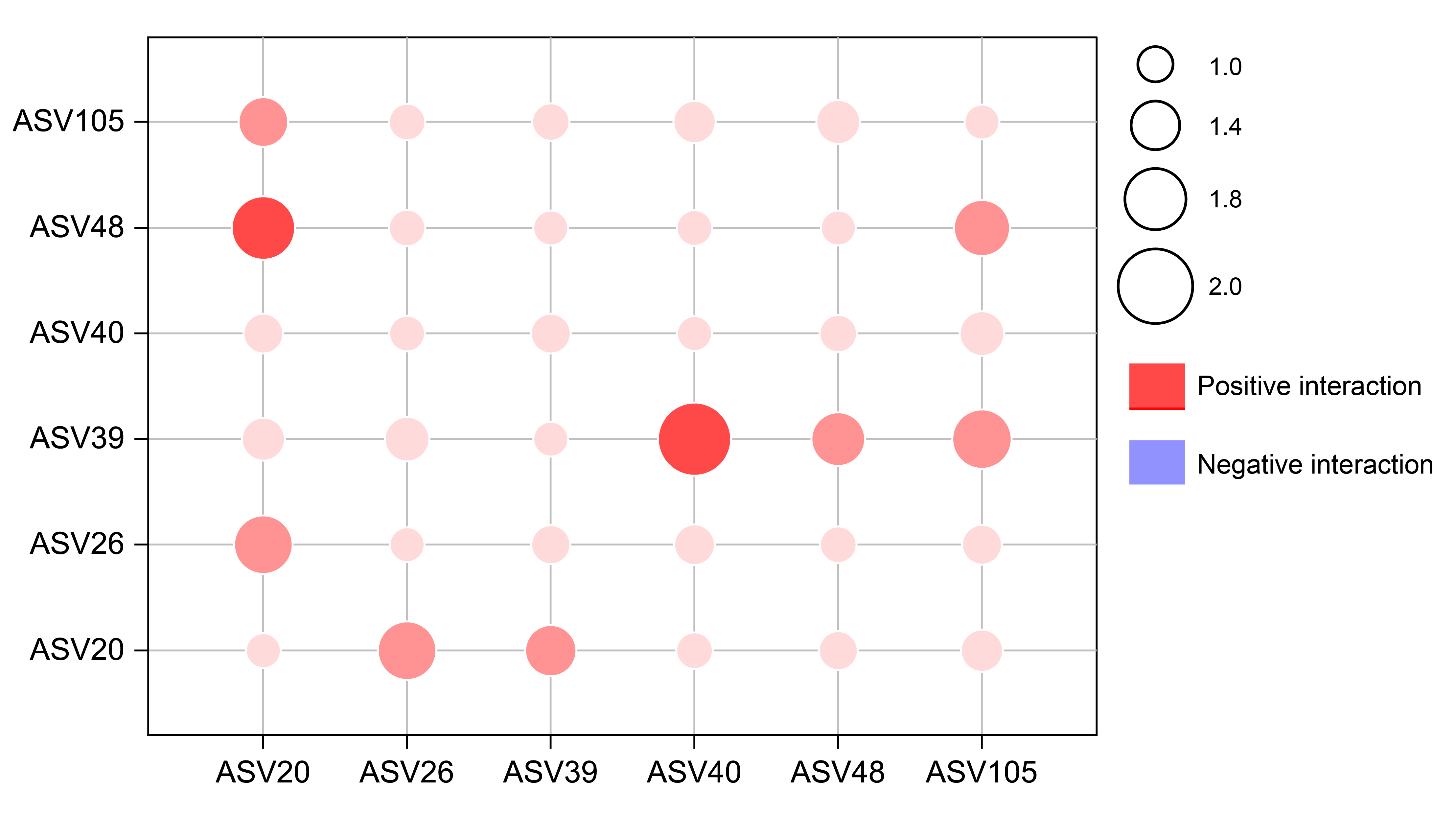


**Supplementary Figure 14. Metabolic interactions among members of the synthetic microbial community.** Different colors represent different interaction modes, with red indicating positive effects and blue indicating inhibitory effects. The size of the bubbles shows the ratio of OD600 values compared to those of strains grown in LB medium.


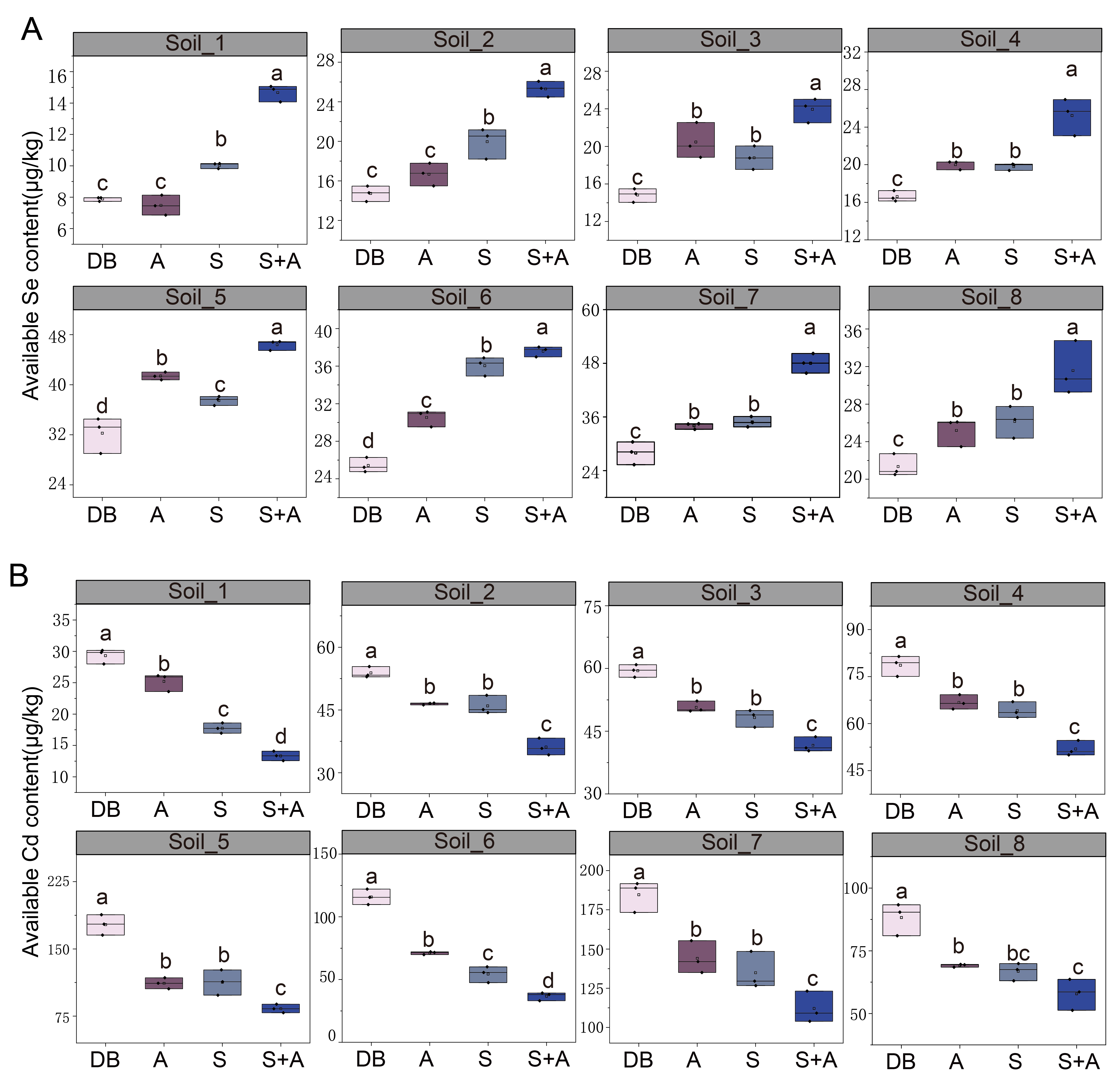


**Supplementary Fig. 15 Soil cultivation validation experiments of glutathione-related amino acids and synthetic microbial communities in eight natural Se- and Cd-enriched soils.** A) Available Se content in soil. B) Available Cd content in soil. ‘A’ represents for the treatment with three amino acids related to the glutathione metabolic pathway added. ‘S’ represents for the treatment with the synthetic microbial community added. ‘DB’ represents the addition of heat-inactivated synthetic microbial communities at a concentration equivalent to the ‘S’ treatment. Different letters indicate significant differences among different treatments at *P* < 0.05 by one-way ANOVA test.


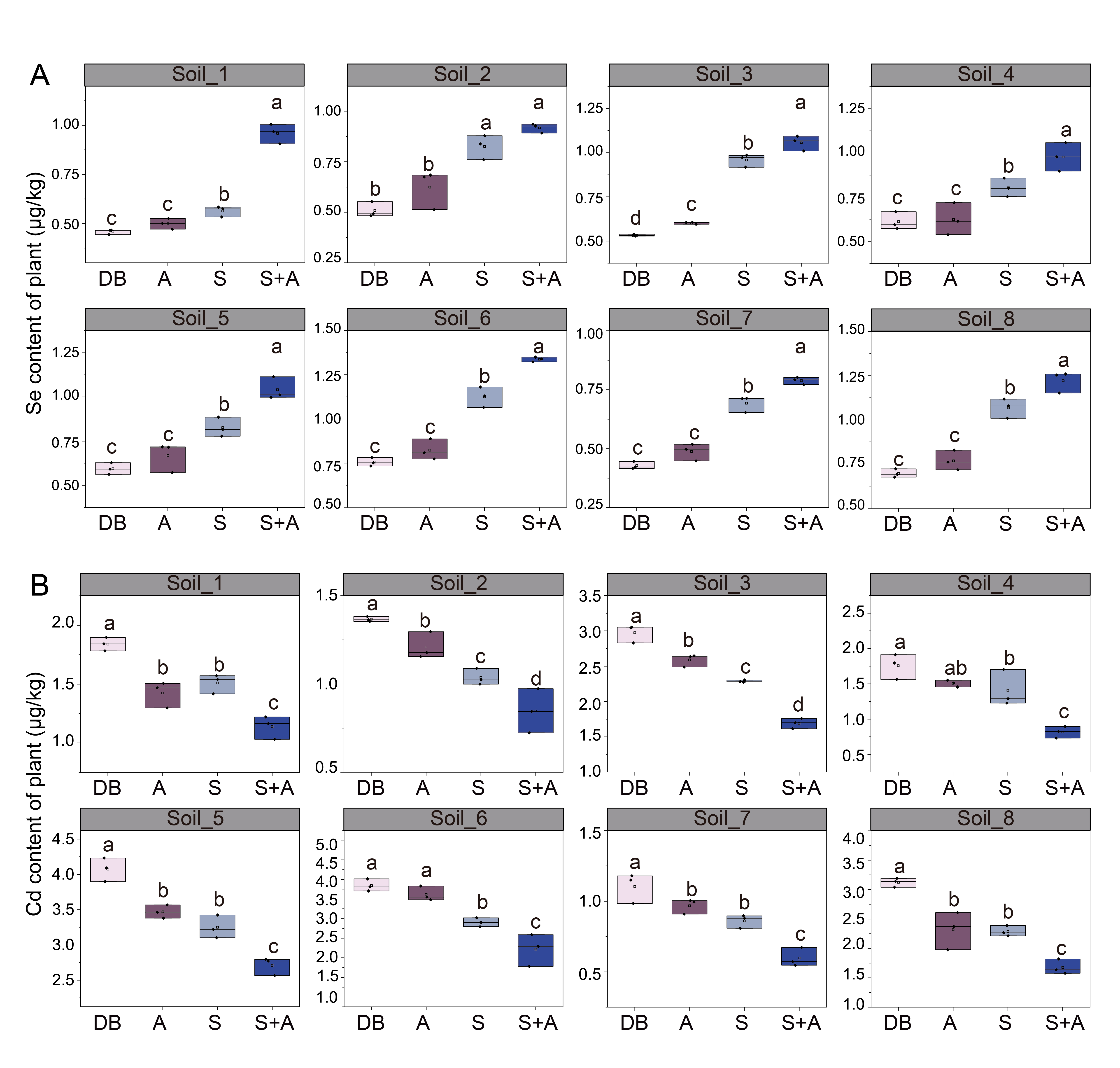


**Supplementary Fig. 16 Pot validation experiments of glutathione-related amino acids and synthetic microbial communities in eight natural Se-Cd rich soils.** A) Se content in plants. B) Cd content in plants. ‘A’ represents for the treatment with three amino acids related to the glutathione metabolic pathway added. ‘S’ represents for the treatment with the synthetic microbial community added. ‘DB’ represents the addition of heat-inactivated synthetic microbial communities at a concentration equivalent to the ‘S’ treatment. Different letters indicate significant differences among different treatments at P < 0.05 by one-way ANOVA test.


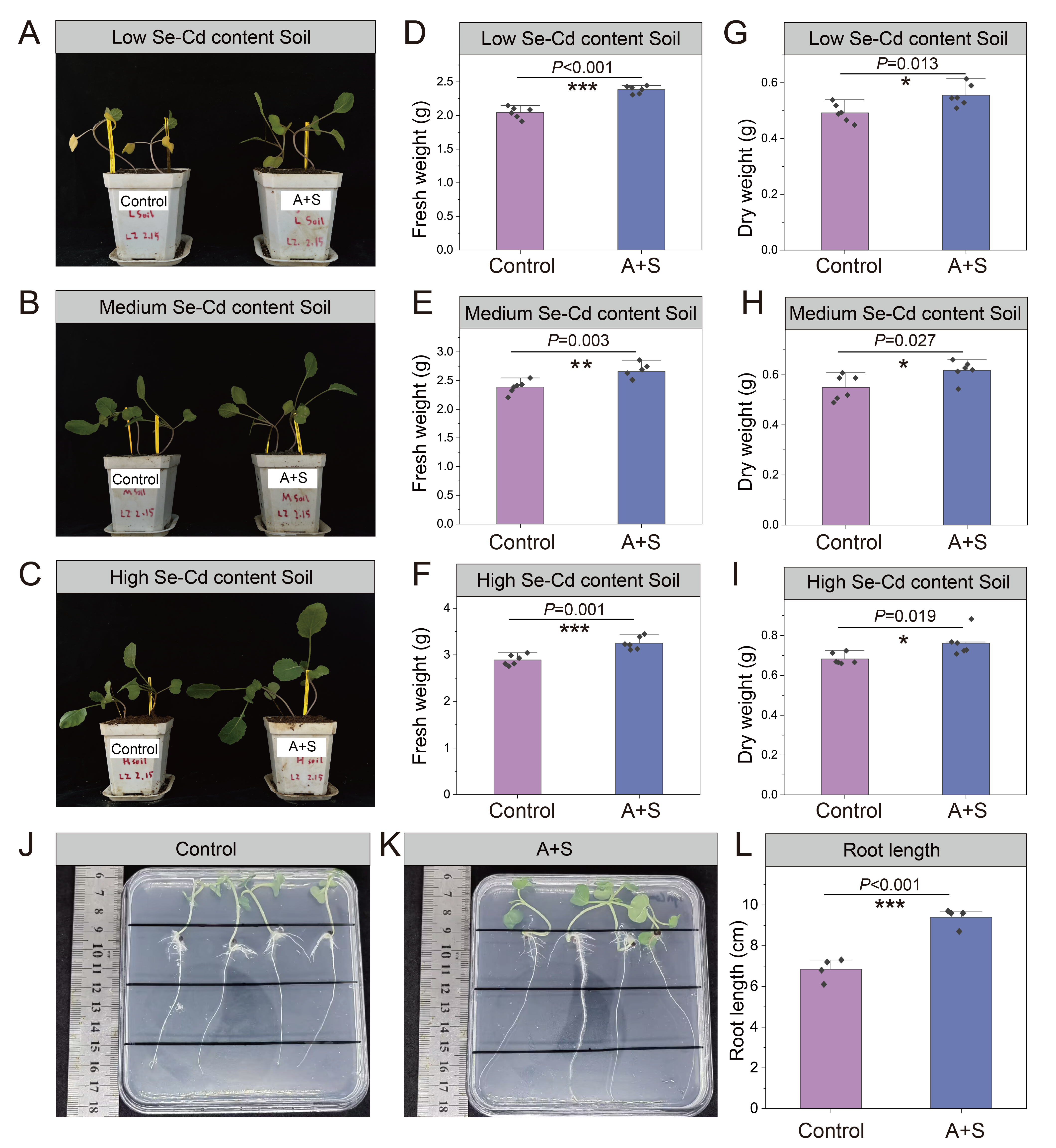


**Supplementary Fig. 17 Synthetic microbial communities and key amino acids related to the glutathione metabolic pathway promote plant growth.** A-C) Effects of applying amino acids related to the glutathione metabolic pathway and inoculating synthetic microbial communities on oilseed rape growth in three soils. (d-i) Effects of applying amino acids related to the glutathione metabolic pathway and inoculating synthetic microbial communities on plant fresh weight (D-F) and dry weight (G-I). J-L) Effects on root length under agar culture. 'A+S' represents the treatment of applying amino acids related to the glutathione metabolic pathway and inoculating synthetic microbial communities. The asterisks represent the level of significance (**P* < 0.05, ***P* < 0.01, and ****P* < 0.001) among different samples based on a one-way ANOVA test with Dunnett’s post hoc analysis.


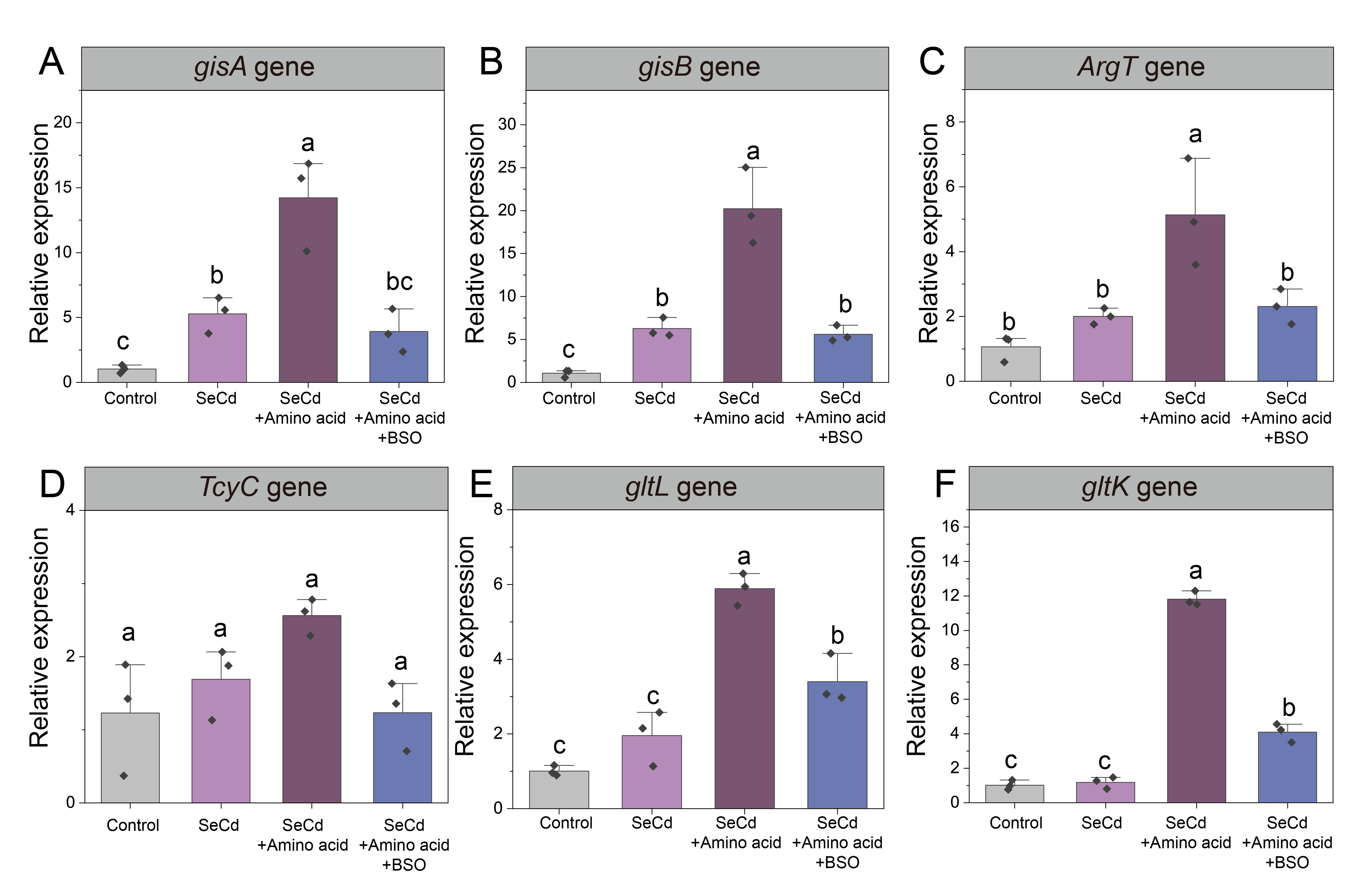


**Supplementary Fig. 18 Relative expression of bacterial glutathione transport genes and amino acid transport genes related to glutathione metabolism.** A-D) The *gisA*, *gisB*, *ArgT*, and *TcyC* genes are related to the transport of glutamate, cysteine, and arginine. E-F) The *gltL* and *gltK* genes are related to glutathione transport. 'Control' means treatment with LB medium only. 'SeCd' means treatment with LB medium containing 0.1 mM Na₂SeO₃ and 0.1 mM CdCl₂. 'SeCd+Amino acid' means adding three amino acids related to glutathione metabolism to the SeCd treatment. 'SeCd+Amino acid+BSO' means adding the glutathione synthesis inhibitor BSO to the SeCd+Amino acid treatment. Different letters indicate significant differences among different treatments at P < 0.05 by one-way ANOVA test.


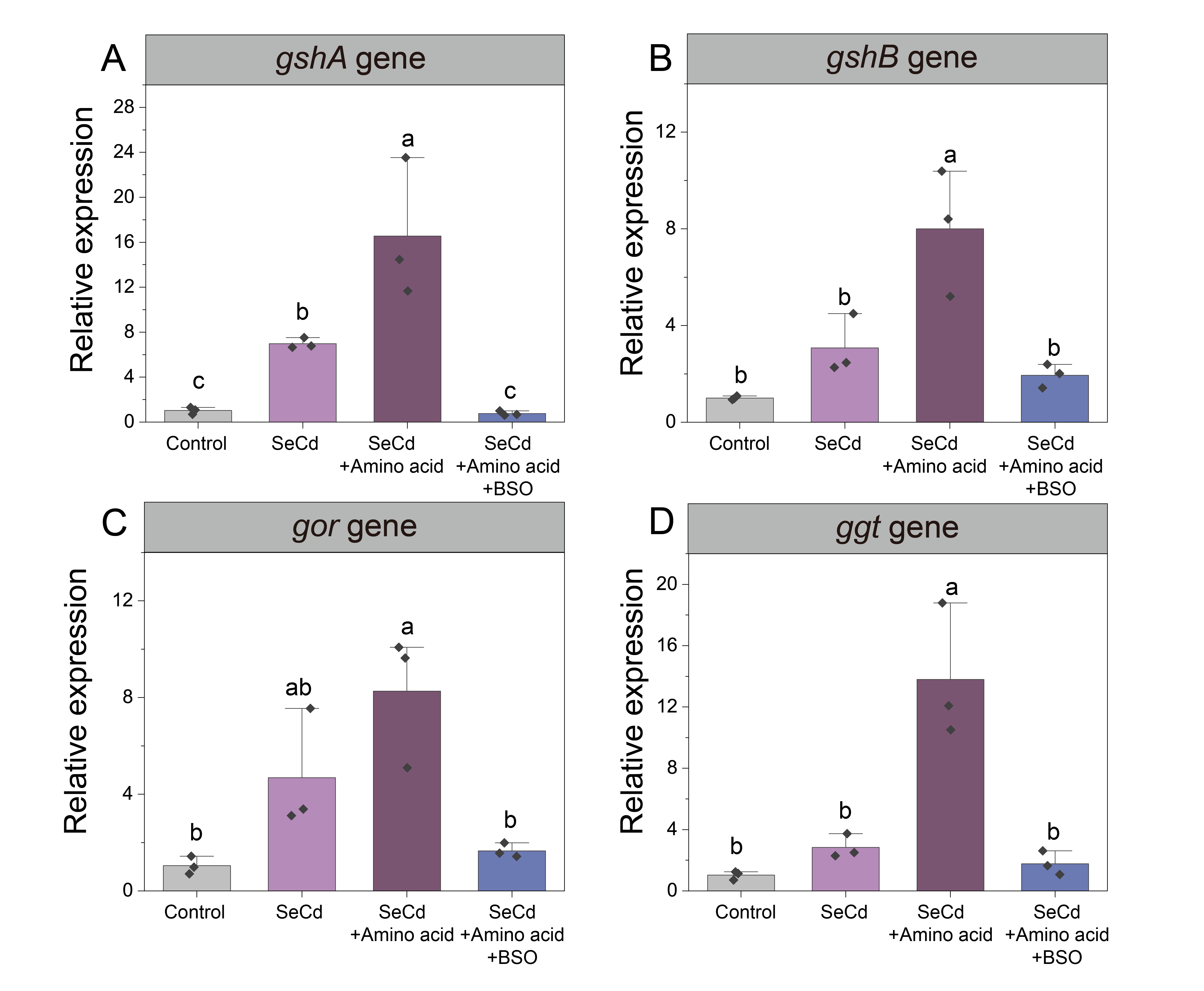


**Supplementary Fig. 19 Relative expression levels of glutathione synthesis and metabolism genes.** A-B) *gshA* and *gshB* are key rate - limiting genes in glutathione synthesis, encoding γ - glutamylcysteine synthetase and glutathione synthetase, respectively. C-D) *gor* and *ggt* are related to glutathione metabolism and redox balance, encoding γ - glutamyltransferase and glutathione oxidoreductase, respectively. 'Control' means treatment with LB medium only. 'SeCd' means treatment with LB medium containing 0.1 mM Na₂SeO₃ and 0.1 mM CdCl₂. 'SeCd+Amino acid' means adding three amino acids related to glutathione metabolism to the SeCd treatment. 'SeCd+Amino acid+BSO' means adding the glutathione synthesis inhibitor BSO to the SeCd+Amino acid treatment. Different letters indicate significant differences among different treatments at P < 0.05 by one-way ANOVA test.

**Supplementary tables**

**Supplementary Table 1 Sampling point coordinates.**

| **Samples** | **longitude** | **latitude** |
| --- | --- | --- |
| Soil_1 | 109.75726°E | 30.33158°N |
| Soil_2 | 109.72862°E | 30.29772°N |
| Soil_3 | 109.85558°E | 30.55328°N |
| Soil_4 | 109.85421°E | 30.57511°N |
| Soil_5 | 109.80382°E | 30.55648°N |
| Soil_6 | 109.4505°E | 30.35821°N |
| Soil_7 | 109.44434°E | 30.35684°N |
| Soil_8 | 109.41673°E | 30.36997°N |
| Soil_9 | 109.40449°E | 30.37638°N |
| Soil_10 | 109.36369°E | 30.37246°N |
| Soil_11 | 109.35004°E | 30.35929°N |
| Soil_12 | 109.29915°E | 30.35764°N |
| Soil_13 | 109.46797°E | 30.09923°N |
| Soil_14 | 109.43079°E | 30.05801°N |
| Soil_15 | 109.43942°E | 30.05106°N |
| Soil_16 | 109.50748°E | 29.94559°N |
| Soil_17 | 109.50814°E | 29.94693°N |
| Soil_18 | 109.58253°E | 30.00509°N |
| Soil_19 | 109.60956°E | 30.02369°N |
| Soil_20 | 109.66867°E | 30.25276°N |
| Soil_21 | 109.68792°E | 30.24475°N |
| Soil_22 | 109.68792°E | 30.24475°N |
| Soil_23 | 109.72944°E | 30.23028°N |
| Soil_24 | 109.52001°E | 30.40129°N |
| Soil_25 | 109.42795°E | 30.35218°N |
| Soil_26 | 109.42795°E | 30.35218°N |
| Soil_27 | 109.39716°E | 30.38472°N |
| Soil_28 | 109.37369°E | 30.36599°N |
| Soil_29 | 109.37369°E | 30.36599°N |
| Soil_30 | 109.35294°E | 30.35643°N |
| Soil_31 | 109.3178°E | 30.34631°N |
| Soil_32 | 109.30956°E | 30.34713°N |
| Soil_33 | 109.30844°E | 30.35158°N |
| Soil_34 | 109.30735°E | 30.35133°N |
| Soil_35 | 109.31943°E | 30.33525°N |
| Soil_36 | 110.02217°E | 30.61828°N |
| Soil_37 | 109.23546°E | 30.25564°N |
| Soil_38 | 109.29204°E | 30.29734°N |

**Supplementary Table 2 Basic physical and chemical indicators of soils with three different selenium and cadmium contents.**

| Soil | pH | AP  content  (mg/kg) | NH_4_^+^-N  content  (mg/kg) | NO_3_^-^N  content  (mg/kg) | OM content  (g/kg) | Total-Se  content  (mg/kg) | Total-Cd content  (mg/kg) |
| --- | --- | --- | --- | --- | --- | --- | --- |
| Low Se-Cd content soil | 4.56±0.03 | 204.89±12.55 | 56.05±2.56 | 1.68±0.11 | 72.65±2.28 | 0.83±0.02 | 0.60±0.03 |
| Medium Se-Cd content soil | 5.11±0.05 | 189.64±8.63 | 62.37±1.64 | 1.43±0.09 | 94.09±3.69 | 2.52±0.08 | 1.41±0.10 |
| High Se-Cd content soil | 4.84±0.06 | 223.27±7.22 | 65.05±3.09 | 1.38±0.12 | 91.03±4.05 | 4.12±0.15 | 2.51±0.18 |

Note: ‘AP’ represents available phosphorus; ‘OM’ represents organic matter.
